# Whole genome regulatory effect of *MoISW2* and consequences for the evolution of the rice plant pathogenic fungus *Magnaporthe oryzae*

**DOI:** 10.1128/mbio.01590-24

**Published:** 2024-09-18

**Authors:** Mengtian Pei, Yakubu Saddeeq Abubakar, Hina Ali, Lianyu Lin, Xianying Dou, Guodong Lu, Zonghua Wang, Stefan Olsson, Ya Li

**Affiliations:** 1 State Key Laboratory of Ecological Pest Control for Fujian and Taiwan Crops, College of Plant Protection, Fujian Agriculture and Forestry University, Fuzhou, China; 2 Key Laboratory for Plant-Microbe Interaction, College of Life Sciences, Fujian Agriculture and Forestry University, Fuzhou, China; 3 Department of Biochemistry, Faculty of Life Sciences, Ahmadu Bello University, Zaria, Nigeria; 4 Shanghai Key Laboratory for Molecular Engineering of Chiral Drugs, School of Chemistry and Chemical Engineering, Shanghai Jiao Tong University, Shanghai, China; 5 Institute of Oceanography, Minjiang University, Fuzhou, China; 6 Synthetic Biology Center, College of Future Technologies, Fujian Agriculture and Forestry University, Fuzhou, China; Harvard Medical School, Boston, Massachusetts, USA

**Keywords:** heterochromatin, epigenetics, nucleosomes, avirulence genes, retrotransposons, evolution

## Abstract

Isw2 proteins, ubiquitous across eukaryotes, exhibit a propensity for DNA binding and exert dynamic influences on local chromosome condensation in an ATP-dependent fashion, thereby modulating the accessibility of neighboring genes to transcriptional machinery. Here, we report the deletion of a putative MoISW2 gene, yielding substantial ramifications on plant pathogenicity. Subsequent gene complementation and chromatin immunoprecipitation sequencing (ChIP-seq) analyses were conducted to delineate binding sites. RNA sequencing (RNA-seq) assays revealed discernible impacts on global gene regulation along chromosomes in both mutant and wild-type strains, with comparative analyses against 55 external RNA-seq data sets corroborating these findings. Notably, MoIsw2-mediated binding and activities delineate genomic loci characterized by pronounced gene expression variability proximal to MoIsw2 binding sites, juxtaposed with comparatively stable expression in surrounding regions. The contingent genes influenced by MoIsw2 activity predominantly encompass niche-determinant genes, including those encoding secreted proteins, secondary metabolites, and stress-responsive elements, alongside avirulence genes. Furthermore, our investigations unveil a spatial correlation between MoIsw2 binding motifs and known transposable elements (TEs), suggesting a potential interplay wherein TE transposition at these loci could modulate the transcriptional landscape of *Magnaporthe oryzae* in a strain-specific manner. Collectively, these findings position MoIsw2 as a plausible master regulator orchestrating the delicate equilibrium between genes vital for biomass proliferation, akin to housekeeping genes, and niche-specific determinants crucial for ecological adaptability. Stress-induced TE transposition, in conjunction with MoIsw2 activity, emerges as a putative mechanism fostering enhanced mutagenesis and accelerated evolution of niche-determinant genes relative to housekeeping counterparts.

**IMPORTANCE:**

Isw2 proteins are conserved in plants, fungi, animals, and other eukaryotes. We show that a fungal Isw2 protein in the rice pathogen *Magnaporthe oryzae* binds to retrotransposon (RT) DNA motifs and affects the epigenetic gene expression landscape of the fungal genome. Mainly ecological niche determinant genes close to the binding motifs are affected. RT elements occur frequently in DNA between genes in most organisms. They move place and multiply in the genome, especially under physiological stress. We further discuss the Isw2 and RT combined activities as a possible sought-after mechanism that can cause biased mutation rates and faster evolution of genes necessary for reacting to abiotic and biotic challenges. The most important biotic challenges for plant pathogens are the ones from the host plants’ innate immunity. The overall result of these combined activities will be an adaptation-directed evolution of niche-determinant genes.

## INTRODUCTION

The eukaryotic genome is organized into condensed nucleosomes, limiting access to DNA for transcription factors and repressors, and more loosely packed regions that are easier to access for interactions with DNA for transcription factors and repressors. The cellular inheritance of such patterns is the basis of animal cell line specialization during embryogenesis (1). Thus, DNA accessibility in fungi also plays an essential role in determining which genes can be efficiently transcribed (2).

Imitation switch 2 (ISW2) belongs to the imitation switch subfamily (Uniprot) of the SNF2 (sucrose non-fermentable)/RAD54 helicase family. ISW2 proteins contain a Myb/Sant domain close to their C-termini that binds DNA. In eukaryotes, especially in plants, Myb-domain-containing proteins usually function as transcription factors (3–26).

A true Isw2 should instead be involved in gene regulation by regulating the access of transcription factors and repressors to the DNA through binding to the DNA and controlling nucleosome positioning, and not as a classic transcription factor (TF) (27–29).

Isw2 proteins have a histone binding domain that interacts with histone 4 of adjacent nucleosomes, and a catalytic ATPase domain closest to the N-terminus that reacts with ATP and changes the Isw2 protein conformation (30–32). The result is that the nucleosome moves toward the Isw2 DNA binding site (28). In that way, Isw2 regulates DNA transcription by changing the access of TFs and repressor proteins to the DNA. Isw2 thus causes a local nucleosome condensation around its DNA-binding sites. As a consequence, a localized nucleosome condensation is created that negatively affects the regulation of the genes at the DNA binding site (30) but favors regulation a bit further away from it (28, 33).

There has been much research into Isw2 protein binding, but it has become clear that *in vitro* mapping of Isw2 binding and nucleosome positioning does not reflect what mechanistically occurs *in vivo* (28, 33), or the very transient nature of the interaction with His4 (34, 35). The interaction is transient in that the nucleosomes closest to the Isw2 binding sites get dynamically positioned by very frequent (seconds) interaction with the Isw2 protein in an ATP-dependent manner. The activity keeps the closest nucleosome(s) almost immobile, while nucleosomes further away get more freedom to move, and the genes there are easier to access and regulate (33). Experiments have shown that both the largest subunits of the Isw2 complex, Isw2, and Itc1 (imitation switch 2 complex subunit 1) (36), are needed for robust, target-specific binding to DNA while Isw2 alone is sufficient for basal-level binding (28).

Furthermore, Isw2 is known to bind DNA preferentially at intergenic regions (30) where transposable elements (TEs) are commonly located (TE target sites). These sites are staggered cut palindromic target sites (37). In addition, TEs are involved in stress adaptation and host specialization in *Verticillium dahliae* (38) as well as in the rice blast fungus *Magnaporthe oryzae* studied here (39, 40) and should especially be needed when the host plant detects the fungus as a pathogen at the transition between endophytic and necrotrophic infection phases of the fungus and thereafter (41).

*ISW2* is highly expressed and often considered a “housekeeping” gene stably expressed under normal conditions (42). The stable expression of housekeeping genes has recently been challenged, and many genes traditionally considered stably expressed are unreliable when expressed outside relatively narrow conditions. Thus better methods for selecting housekeeping genes that better fit a specific set of conditions or tissues have been employed (43). On the other hand, the change in ratios of target genes to housekeeping genes reflects the actual change in gene expression ratios of 2 genes of interest. In a previous paper from our lab (44), we used correlations of expression profiles between genes of interest to probe possible gene functions. That can be done in transcriptomic data obtained from many labs and is especially useful if the calculations are done for individual data and not just the average value of replicates. Then, variations between replicates can be used to calculate the correlation between any two genes’ expressions since these ratios reveal the dependencies of one gene’s expression on the other, or both genes’ expressions on a third one. Thus, the ratios of gene expressions can reveal putative gene functions and help identify genes with specific putative functions that should correlate with the expression of well-known orthologous genes (44).

In this study, we investigated the putative *MoISW2* gene in *M. oryzae* as a true *ISW2* gene that affects gene regulation close to the MoIsw2 protein binding sites in the fungal genome. Deletion of the gene negatively affected the fungal pathogenicity and stress tolerance. We further showed that MoIsw2 ATP binding is at TEs within the genome and that genes close to the binding sites are more differentially regulated (negatively or positively). The genes that get increased access are mainly niche-determinant and oxidative phosphorylation-related genes. Genes with decreased access are DNA synthesis genes, ribosome genes and other genes linked to fast growth. That combined with oxidative stress-induced TE transpositions, changing the binding sites of MoIsw2 makes the binding of the protein to TE sites and its activities a likely key mechanism behind biased evolution (45) in *M. oryzae* with a faster mutation rate for affected niche-determinant genes.

## RESULTS

### Bioinformatics similarity and domain structure

**Fig 1:**
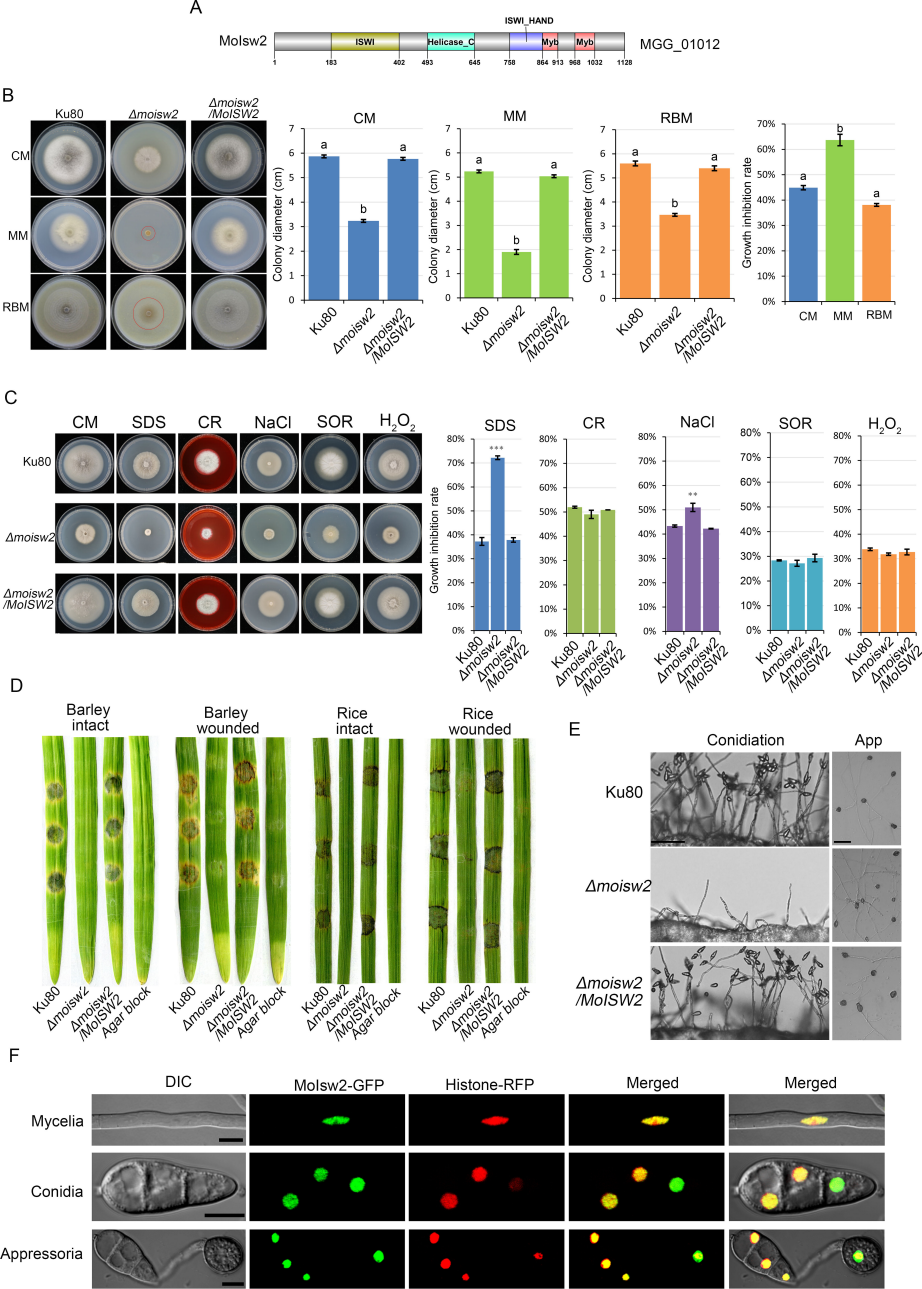
Domain structure, knockout phenotypes compared to the background *Ku80*, and localization of MoIsw2. (**A**) Domain structure. (**B**) Growth phenotypes and growth on complete medium (CM), minimal medium (MM), and rice bran medium (RBM). (**C**) Stress phenotypes on CM with added sodium dodecyl sulfate (SDS), Congo Red (CR), salt (NaCl), sorbitol (SOR), or hydrogen peroxide (H_2_O_2_). (**D**) Infection phenotypes on barley and rice leaves. (**E**) Conidia formation and appressoria formation from hyphae. (**F**) Subcellular localization in mycelia, conidia, and appressoria MoIsw2-GFP localizes to nuclei (Histon1-RFP nuclear marker). Size bars in panel **F** are 5 μm long. Error bars are 95% confidence intervals and bars with non-overlapping error bars are significantly different (*P*_same_ < 0.05). The same letter on the bars in panel **B** indicates *P*_same_ > 0.05. The stars above bars (**C**) indicate significant differences from the *Ku80* controls; *, *P*_same_ < 0.05; **, *P*_same_ < 0.01; ***, *P*_same_ < 0.001.

In *M. oryzae* the protein encoded by the MGG_01012 gene is annotated as a putative MoIsw2 in the NCBI database. From its N to C termini, an Isw2 protein consists of Dexx (ISWI), helicase C, HAND (ISW_Hand), SANT (Myb), and SLIDE domains (46). The putative MoIsw2 protein was phylogenetically compared with other Isw2 orthologues in closely related fungi as well as in more distant fungi, animals, and a protist; and the proteins were found to be well conserved, both in sequence and length from the most distant organism Eukaryote the model protist *Dictyostelium discoideum* (Fig. S1A and B). A phylogenetic tree was constructed that roughly seems to mirror the evolution of Eukaryotes (Fig. S2). In mammals, there are two ISW2 orthologues, SMARCA1 and SMARCA2 (SWI/SNF related, matrix associated, actin dependent regulator of chromatin, subfamily a) (47), and the MoIsw2 has the most similarity with the ubiquitous fungal/metazoan version (e.g., SMARCA2). Isw2 orthologues from *Gallus gallus* (hen), *D. discoideum*, and *M. oryzae* in the far ends of the phylogenetic tree were compared for domain structure with the domain structure of the well-studied Isw2 in *Saccharomyces cerevisiae* (baker’s yeast) (48) and the protein domain structure (domains types and order of domains) is conserved in all cases (Fig. S3). Thus, the MoIsw2 protein is most probably an Isw2 with the typical Isw2 domain structure shown slightly simplified (Fig. 1A).

### Phenotypes of Δ*Moisw2* mutant and subcellular localization of the MoIsw2-GFP protein

The *MoISW2* gene was deleted in the *M. oryzae Ku80* background strain and the deletion was confirmed by Southern blot (Fig. S4). The effect of this mutation on the vegetative growth of the fungus was tested on three types of media, namely, complete medium (CM), minimal medium (MM), and rice bran medium (RBM), after incubation at 28°C for 10 days. The assays showed a significantly decreased colony size of the mutant on all three media when compared to the background strain *Ku80* and the complemented strain (Δ*Moisw2*/*MoISW2*) (Fig. 1B).

The possible role of the *MoISW2* gene on stress resistance was tested by growing the various strains on CM media supplemented with different stress-inducing agents (49). The CM medium without the addition of any stress-inducing agent was used as the control medium. The Δ*Moisw2* mutants are strongly inhibited by both SDS affecting membrane integrity and NaCl that induces osmotic and ionic stresses (Fig. 1C), suggesting the involvement of the MoIsw2 in withstanding osmotic and ionic stresses in *M. oryzae*. Congo red (CR) with effects on cell wall organization, sorbitol (SOR; an osmolyte), and hydrogen peroxide (H_2_O_2_, an oxidative stress inducer had no substantial influence on the *in vitro* vegetative growth of the mutant.

Furthermore, we tested the ability of the mutant to produce conidia, which are essential for the spread of rice blast disease from plant to plant in the field. We tested this by checking the abundance of conidiophores (which carry the conidia) in the various strains. While the WT and complemented strains produced many conidiophores, each bearing many conidia, the Δ*Moisw2* mutant only produced scanty conidiophores bearing few or no conidia (Fig. 1E).

Next, the Δ*Moisw2* mutant was tested for pathogenicity on rice and barley leaves and compared with the *Ku80* background strain and Δ*Moisw2*/*MoISW2* complemented strain. Since the Δ*Moisw2* mutant does not produce enough conidia to be used for inoculation, agar plugs were used to infect healthy rice and barley leaves in the infection assay. The assay still showed no infection for the Δ*Moisw2* mutant strains (Fig. 1D). We reasoned that this could be due to an inability of the mutant to penetrate the plant cuticles. Therefore, we inoculated the fungal strains on aseptically wounded leaves and found that the Δ*Moisw2* mutant could still not develop any obvious disease symptoms on the leaves, indicating that *MoISW2* is needed for any successful infection of rice or barley leaves (Fig. 1D). From hyphae, *M. oryzae* can form appressoria needed for leaf infection. We found, however, that dark appressoria formation was not affected by the *MoISW2* mutation, indicating that the cause of no infection in the infection assay was probably not a complete lack of appressoria formation (Fig. 1E).

Finally, the Moisw2/*MoISW2-GFP* complementation was used to visualize the subcellular localization of MoIsw2. This was performed in a strain expressing histone 1-mCherry (a red fluorescent protein [RFP]) (as a nuclear marker) (50) because we expected that MoIsw2-GFP should accumulate in the nucleus as its orthologues do in other eukaryotes. The MoIsw2 protein was observed to consistently colocalize with the nuclear marker in hyphae, conidia, and appressoria (Fig. 1F).

Taken together, these results suggest that MoIsw2 is important for the development, stress resistance, and virulence of *M. oryzae*, and it localizes to nuclei, consistent with its potential role in regulating gene expression as an Isw2 protein.

### Correlation analyses of *ISW2* expression with expressions of nucleosome histones and ITC1

**Fig 2:**
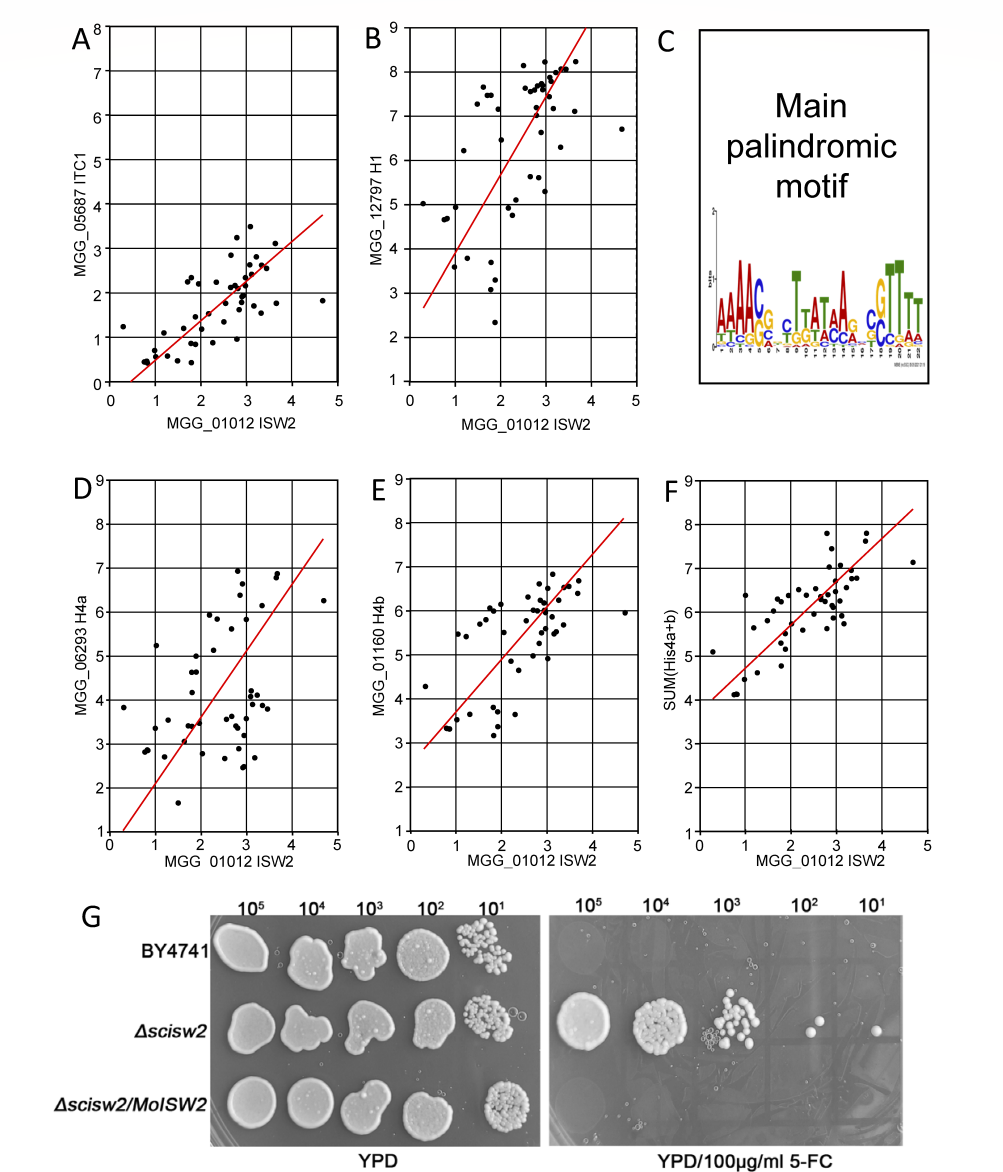
Log2 reduced major axis (RMA) correlations of a putative *ITC1* (**A**), the linker histone *HIS1* (**B**), and the two putative *HIS4* (**D–F**) genes putatively expressing the same protein known to interact with the expression of the putative *MoISW2* (*x*-axis) in published RNAseq data at different stages of plant infection. Each dot represents the two genes’ expression in a separate RNAseq data set. RMA fitting was used to handle the problem of errors in both *x* and *y* axis values, an inherent consequence of plotting the expression of 2 genes against each other. Each dot corresponds to the value from one transcriptome. (**A**) ITC1, *P*_(uncorrelated)_ = 1.57E−7; (**B**) *HIS1*, *P*_(uncorrelated)_ = 1.48E−5; (**D**) *HIS4a P*_(uncorrelated)_ = 0.0044; (**E**) *HIS4b P*_(uncorrelated)_ = 3.81E−7; (**F**) *HIS4a+4*b since both *HIS4* genes are predicted to encode for the same protein, *P*_(uncorrelated)_ = 5.82E−10. Also, note that panels D and E have been positioned so that the effect of the addition log2(His4a + His4b) can be seen visually. All plots are shown with equal *x* and *y* scale gradings so that it is easy to compare slopes between graphs visually. (**C**) The most frequently occurring palindromic motif was found in 196 sequences in the chromatin immunoprecipitation sequencing (ChIP-seq) data using MoIsw2-GFP as bait. (**G**) *MoISW2* could restore the sensitivity of a yeast ISW2 mutant Δ*scisw2* to 5-fluorocytosine. *S. cerevisiae* wild-type strains BY4741 and ISW2 mutant Δ*scisw2* were used.

Histone 1 (*HIS1* gene) is involved in epigenetic activities by changing access to DNA, and its expression is linked to silencing activity (51). The active Isw2 protein complex contains Itc1 as a subunit, binds to DNA, and interacts with histone 4 in the closest nucleosome (33) affecting local access to DNA. We used published expression data from many experiments in Guy11 (the WT of *Ku80*) recorded from the course of rice leaf infection to investigate correlations between the putative *MoISW2* and the MoIsw2 complex gene candidates in the same way as we have done before for other genes using the same data (44). *MoISW2* should be transcriptionally co-regulated with the genes encoding other subunits of the MoIsw2 complex, as well as with MoHis1 which is generally needed for epigenetic silencing. In *M. oryzae,* MGG_06293, and MGG_01160 are two *HIS4* genes predicted (NCBI) to encode identical His4 protein. We call these genes *MoHIS4a* and *MoHIS4b,* respectively. Thus, the sum of these gene regulations should best reflect the regulation of the MoHis4 protein. Isw2 further works together with ITC1 in a complex that interacts with His4 (27). Thus, the expression of these four putative genes (*MoISW2*, *MoITC1*, *MoHIS4a,* and *MoHIS4b*) which produce three proteins (MoIsw2, MoItc1, and MoHis4), should be correlated in *M. oryzae,* and they are well correlated in the data from plant infection (Fig. 2A, D, and E; Fig. S5). Also correlated with *MoISW2* expression is the *MoHIS1* gene (Fig. 2B). The closest correlation of *MoISW2* expression is even with the sum of the expression of the two MoHis4 genes (i.e., *MoHIS4a + MoHIS4*b) (Fig. 2F).

Taken together with sequence and domain similarities (Fig. S1 to S3), this suggests that the putative MoIsw2 is a true Isw2, as the proteins encoded by these genes interact with His1, Itc1, and His4 in a complex (28). Furthermore, the expression of *MoHIS2B* is related to the overall growth rate (and thus *de novo* DNA synthesis rate) (44) and is correlated with *MoISW2* but not with a steep slope (Fig. S6C). This suggests that a lower amount of MoHis2B than MoHis4 is required for handling the *de novo* DNA synthesis when Isw2 activity is high. This in turn could mean that the DNA-synthesis rate decreases with higher *MoISW2* expression. Furthermore, the expression of *MoHIS3*, but not *MoHIS2A*, is significantly correlated with the putative *MoISW2* (Fig. S6A and B). Although the MoIsw2 protein has been reported in the literature to interact with His4, such interactions could not be detected using a yeast two-hybrid assay (data not shown) and the lack of positive results in this assay is likely due to the transient nature of the interaction (34, 35).

To further verify that MoIsw2 is an orthologue of yeast ISW2, the *MoISW2* was transformed into the yeast ISW2 mutant Δ*scisw2* and tested whether *MoISW2* could restore the phenotype of the *ScISW2* gene. Previous work has indicated that a *Cryptococcus neoformans* ISW1 mutant (only one ISW orthologue in that fungus as in *M. oryzae*) displayed reduced sensitivity to the antifungal agent 5-fluorocytosine (5-FC) (52). Given the high similarity (*P* = 0.0, with 85% cover, 58% identity, in an NCBI blast) between *MoISW2* and *CnISW1*, we used 5-FC in our study to assess whether expressing *MoISW2* in the yeast mutant could restore the sensitivity of *ScISW2* to 5-FC. As illustrated in (Fig. 2G) the transformant Δ*scisw2/MoISW2* restored the sensitivity to 100 µg/mL 5-FC to become similar to the yeast WT BY4741.

Taken together, the putative *MoISW2* gene likely encodes a putative MoIsw2 protein that is involved in targeted local chromatin compaction in collaboration with MoHis1, MoItc1, and MoHis4 (28, 33, 51). From now on, we will omit “putative” as an attribute and investigate whether MoIsw2 has the expected functions as an Isw2 and what other effects it has on the biology of the fungus. Furthermore, the local chromatin compaction and the regulatory effect caused by MoIsw2 should be dynamic and stronger the more the gene and its encoded protein are expressed, as well as the more ATP is available for the dynamic interaction.

### ChIP-seq analysis of MoIsw2 to find DNA binding motifs

We generated a MoIsw2-GFP fusion protein to perform a chromatin immunoprecipitation sequencing (ChIP-seq) analysis to find conserved DNA binding palindromic motifs for the binding of MoIsw2 to *M. oryzae* DNA sequences. As mentioned in the introduction, Isw2 proteins are known to preferentially bind DNA in intergenic regions where transposable elements (TEs) are frequently located (30) with staggered palindromic motif target sites (37). *M. oryzae* TEs are mainly intergenic (39, 53) and affect secreted protein expression important for pathogenesis differently in different *M. oryzae* strains (53).

Data from our lab (53) combined with the present data showed hits mainly for intergenic sequences (Supplemental data 1 and 5, downloadable through Table S2). where the TEs are preferentially located (37). For these reasons, we searched for palindromic motifs in the MoIsw2-GFP ChIP-seq sequences. We used as input the sequences (peaks) from the ChIP-seq analysis and used the MEME website to find common DNA binding motifs. In addition, we searched the ChIP-seq sequences for previously identified TE sequences (53).

**Fig 3:**
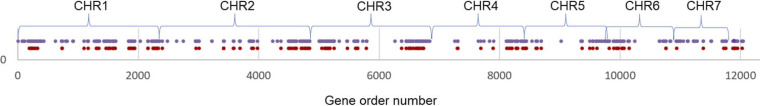
The position of TEs and identified palindromic MoIsw2 binding sites in DNA largely overlap along the whole genome. Purple dots mark the position of the identified TE positions present in the ChIPseq and red dots the positions of the MoISW2 palindromic motif sites.

Three palindromic motifs were found (Supplemental data 2, downloadable through Table S2) with 47, 196, and 32 occurrences for motifs 1, 2 (Fig. 2C), and 3, respectively, located at 267 DNA locations in total. Most of these locations are intergenic and only a few ChIP-seq sequences had more than one motif. Further analysis of the motif with 196 hits (motif 2) revealed that most of these hits were indeed intergenic (113 intergenic locations), while the rest (54) were in the promoter region of a gene. Using a TOMTOM search at the MEME website, we found that motif 2 has similarities with a known human Myb-protein containing a DNA binding motif (Fig. S7; Supplemental data 3, downloadable through Table S2).

### MoIsw2 DNA binding and its influence on nearby avirulence gene expression

Transposable elements interacting with MoIsw2 may play a direct role in a pathogen-plant arms race if they affect the expression of the nearby avirulence genes (39, 53) through targeted nucleosome condensation (55–57). Avirulence genes are often effectors a pathogen needs to efficiently cause disease and parasitize its host (58). As such, avirulence genes should preferably accumulate adjacent to the MoIsw2 binding site during recent evolution, and their expression should be affected differently depending on their closeness to the MoIsw2 binding site. To test this we first made a list of all known avirulence genes in *M. oryzae* at the NCBI database (Table 1). We found 16 avirulence genes of different types and in addition an avirulence gene cluster of 12 genes for a cytochalasan-type compound biosynthesis (59, 60). We then made an RNAseq of the background strain *Ku80* and compared it with the *MoISW2* mutant which also could be compared with the downloaded data. Two other avirulence genes, Avr-PWL1 and Avr-PWL2 (61), together with the 16 Avr genes, make 18 classic avirulence-type genes, excluding the cytochalasan gene cluster genes (Table 1). Most of the genes in the cytochalasan cluster are positioned close to the MoIsw2 palindromic DNA binding motif sites, and most of them are differently expressed in strains *Ku80* and 98-06 (Table 1), which might lead to the production of different final metabolites from the cytochalasan gene cluster even if the different metabolites function as virulence factors.

**TABLE 1:** Comparison of the position of avirulence genes compared to the position of the MoIsw2 palindromic DNA binding motif sites in strain Guy11, as well as differential regulation between experiments of the avirulence genes in MoISW2 knockout compared to the background, and differential regulation of the same genes during infection of rice in strain Guy11, and strain 98-06 from published data^*a*^^,^

ID	Annotation	Distance from the closest gene to MoIsw2 binding site Guy11	DelR Guy11	Guy11 expr (X)	98-06 expr (X)	VarR Guy11
MGG_12447	Cytochalasan	0	19.31	**X**		48.20
MGG_08386	Cytochalasan	NP				
MGG_08377	Cytochalasan	NP				
MGG_08378	Cytochalasan	1	15.25	X		15.25
MGG_08380	Cytochalasan	?			**X**	
MGG_08381	Cytochalasan	?			**X**	
MGG_08384	Cytochalasan	2	0.08	X	X	**29.55**
MGG_08389	Cytochalasan	2	2.33	X	X	**24.59**
MGG_08390	Cytochalasan	1	0.00	X	X	**56.00**
MGG_08391	Cytochalasan	?			**X**	
MGG_15927	Cytochalasan	?			**X**	
MGG_15928	Cytochalasan	NP				
	Avirulence genes					
MGG_07199	ATR13, RxLR effector	170	0.04	X	X	7.27
MGG_10556	Avr_Pii	NP				
MGG_17614	Avr_Pii	NP				
MGG_13283	Avr_Pik	NP				
MGG_15972	Avr_Pik	?			**X**	
MGG_03029	Avr_Pita1	175	37.08			19.74
MGG_07038	Avr_Pita1	169	2.00			6.25
MGG_09617	Avr_Pita1	NP				
MGG_10927	Avr_Pita1	20	0.07			**24.00**
MGG_03808	Avr_Pita1 like	NP				
MGG_15370	Avr_Pita1 like	1	0.28			**56.00**
MGG_17611	Avr_Pita1	NP				
MGG_14981	AVR_PiTA2	NP				
MGG_15212	AVR_Pita2	?			**X**	
MGG_18041	Avr_Piz-t	NP				
MGG_03685	Avr-Pi54	238	14.11	**X**		3.86
MGG_13863	Avr-PWL1	?			**X**	
MGG_07398	Avr-PWL2	NP				
Sum number				7	11	
Only in one of the strains				3	7	
In both				4		

^*a*^ Bold underlined Xs mark genes that MoIsw2 helps regulate only in Guy11. Bold Xs mark genes that are only regulated during infection by 98-06. DelR and VarR contain variation in expression in RNAseq data between mutant and Ku80, and between 55 downloaded RNAseq data sets from different labs, respectively (see text for explanation of these measures). Several of the listed avirulence genes are not present in any of the two strains and some are only present in one of them. NP, not present in the NCBI data for Guy11 or 98-06, Bold numbers, seem to be more important during plant infection. ?, present in Guy11 but in a contig that is not ordered to the described chromosomes in the BROAD data; thus, the distance to the closest MoIsw2 binding site is unknown.

Five other avirulence genes (MGG_03029, MGG_07038, MGG_10927, MGG_15370, MGG_03685) are very little expressed in both strains even if VarR and/or DelR is high (Table 1). Of these five genes, two genes (MGG_15370 and MGG_10927) are close to the MoIsw2 binding site, and the other three are more distant in Guy11(*Ku80*). Two avirulence genes (MGG_17614 and MGG_15611) are in supercontigs with unknown gene order in our data, so it is unsure how far they are from an identified MoIsw2 binding site.

### TE position and MoIsw2 DNA binding

We searched the short MoISW2 ChIPseq DNA sequences for described TEs sequences (53) and found them in 92.2% of sequences while the larger genome outside these ChIPseq sequences is almost devoid of TEs (Supplemental data 5, downloadable through Table S2). We mapped the MoIsw2 binding sites to the genome as well as the sites of all found TEs (Fig. 3). These data and analyses with additional references are available in supplemental data files (Supplemental data 4 and 5, downloadable through Table S2). In conclusion, MoIsw2 and retrotransposon (RT) activities can together be instrumental in creating different regulatory landscapes in strains of *M. oryzae*.

### MoIsw2 regulates the genes closest to the MoIsw2 motif sites

**Fig 4:**
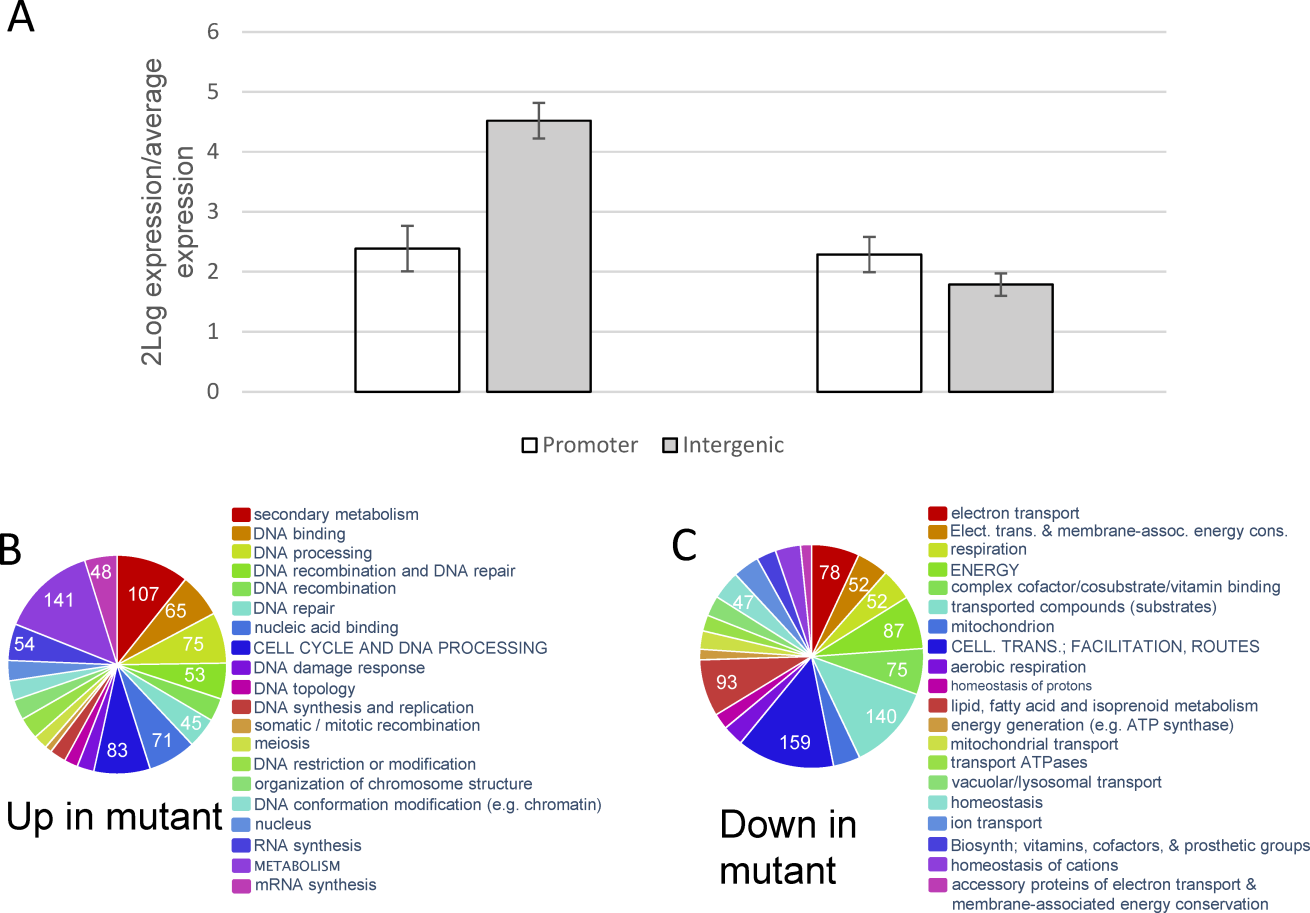
Deletion-induced change in expression of genes next to the predicted palindromic MoIsw2 binding site with hits for the MoISW2 binding in the ChIP-seq data compared to the average regulation of all genes. (**A**) Normalized expression of genes with a binding site in the promoter region and the closest gene when MoISW2 is intergenic. Deletion of MoISW2 upregulates both gene types and upregulates genes slightly further away from the binding site, while the absolute (plus or minus) regulation is more equal for both types of genes. Error bars indicate a 95% confidence interval using a non-parametric bootstrap procedure in PAST that works well for non-Gaussian distributions (BC*a =* Efron’s non-parametric bias-corrected and accelerated procedure was used to correct for eventual bias in the data) (promoter N = 63, intergenic N = 112). Bars with non-overlapping error bars have a *P*_same_ < 0.05. (**B**) Twenty most significantly enriched upregulated FunCat gene categories in the *DMoisw2* compared to the background strain *Ku80*; secondary metabolism, DNA-binding, and genes for DNA-related activities and synthesis (anabolism). (**C**) Twenty significantly enriched downregulated genes in the mutant are genes for, mitochondrial activities like electron transport and mitochondrial biosynthesis, respiration, and transport routes (catabolism).

Next, we compared the RNAseq data for the Δ*moisw2* mutant strain and the background *Ku80* strain for overall gene regulations. There was a general upregulation of the genes closest to the MoIsw2 binding sites in the Δ*Moisw2* mutant (Fig. 4). For these genes, the Δ*Moisw2*/*Ku80* expression ratio is lower when the MoIsw2 is predicted to bind directly in the promoter region than intergenic (Fig. 4A left two bars). On the other hand, if absolute (positive or negative) regulation was considered both types of genes, with MoIsw2 binding in the promoter or intergenic region, had similar absolute regulation indicating that many genes having MoIsw2 binding in their promoter regions are indeed repressed in Δ*Moisw2* (Fig. 4A right two bars). This suggests that without MoIsw2 activity, repressors can also get access to the DNA (Fig. 4A right). Our observations support that MoIsw2 functions as an Isw2 protein that creates a local nucleosome condensation at specific nucleosomes (33) that should also increase transcriptions/repressions of the genes at slightly further distances from the DNA MoIsw2 binding sites.

### Overall functional classification of differentially expressed genes in the RNA-seq data

In the Δ*Moisw2* mutant grown on MM-medium, 339 genes were significantly upregulated relative to the background *Ku80*. The two largest significantly overrepresented gene categories were those related to secondary metabolism and DNA-binding, while most other categories are related to DNA synthesis, DNA-linked activities, and other growth-related processes (anabolism) (Fig. 4B). These are the genes MoIsw2 likely negatively regulates in the background *Ku80*. Most downregulated genes in the Δ*Moisw2* are genes involved in mitochondrial electron transport, other mitochondrial processes, transported compounds, and other processes connected with uptake and respiration and oxidative phosphorylation (catabolism) (Fig. 4C).

Our results thus indicate that MoIsw2 is involved in regulating the balance between anabolism and catabolism.

### Local gene regulations close to the MoIsw2 palindromic DNA binding sites fit the Isw2-specific targeted DNA binding model

DNA is wrapped around and slides around nucleosomes of similar sizes, with a constant overall nucleosome repeat length (NRL) (33, 51, 54, 62) and NRL’s sizes vary slightly depending on the organism, cell type, and cell status (62).

To further investigate local regulation at or close to the binding sites, we revisited our RNAseq data and aligned the MGG codes based on the order of the genes on the chromosomes (chromosome order downloaded from BROAD).

Dynamic changes due to chromosome packing affected by MoIsw2 should result in the same regulatory landscape in our deletion experiment compared to the background as in the downloaded data [comparing different RNAseq sets for Guy11(*Ku80*)]. Since MoIsw2 activity mainly affects the regulation of respiratory catabolism activity (nutrient availability in the niche) as mentioned above, the largest variation in expression should be for genes positioned close to the MoIsw2 binding sites in the DNA. The change of the measured positive or negative gene expression comparing Δ*Moisw2* mutant strain with the background *Ku80* (DelR) could then be compared with the variation in gene responses (positive and negative) in the downloaded 55 RNAseq data sets from several labs (VarR). Similar regulatory landscapes should be found if MoIsw2 is instrumental in forming the WT landscape in Guy11(*Ku80*). We found that the two gene expression measurements were log-log correlated (*P*_uncorrelated_ = 5.3E−196) and that the response slope of the correlation was 0.56 with VarR on the x-axis and Del R on the *y*-axis (Fig. S8).

**Fig 5:**
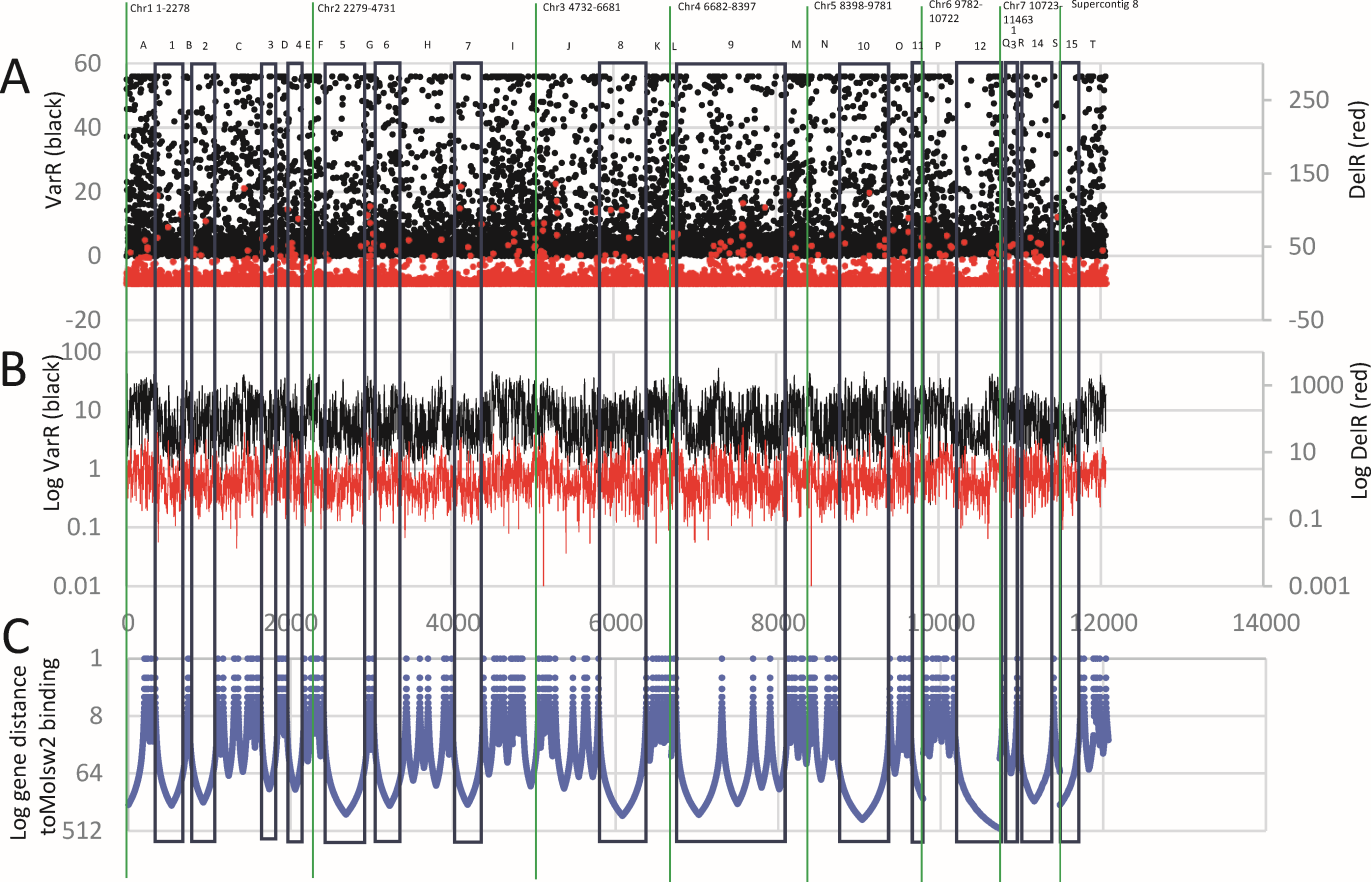
VarR and DelR along the chromosomes compared to, and in relation to, found MoIsw2 1,2,3-palindromic DNA motifs. (**A**) VarR (black dots) and DelR (red dots). (**B**) Same data as in panel A but with log scales. Shows that VarR and DelR show similar regulatory profiles indicating that the VarR regulatory profile is caused by MoIsw2. (**C**) Distance of genes to the closest MoIsw2 binding motif. Note that the distance is reverse and logarithmic. For panels A–C the *X*-axis corresponds to gene order in the genome from the first gene to the last on each chromosome and in chromosome order 1–7. A–T mark suggested genomic regions with high variability of gene expression that are close (less than 16 genes away) to the Isw2 1,2,3-motifs binding sites, and 1–15 mark genomic regions with lower variability.

We further calculated the gene distance between each gene and the closest MoIsw2 binding site by mapping the ChIPseq sequences to the gene positions on the chromosomes, plotted the distance together with plots of the gene expression change from our experiment (DelR) and the variation in the downloaded data (VarR) along all chromosomes (Fig. 5). From these comparisons, one can see that gene regulatory responses along the genome are dependent on MoIsw2 and its binding to DNA. Furthermore, we can identify regions along the DNA with high VarR and DelR. Genes in these regions are less than 16 genes distant from the closest MoIsw2 DNA binding site (Fig. 5, regions A–T) and regions with low VarR and DelR 17 to about 500 genes distant from the MoIsw2 binding motifs (Fig. 5, regions 1–15). In total 2,448 genes of the total 11,557 genes investigated (25%) are in regions with high VarR and DelR.

**Fig 6:**
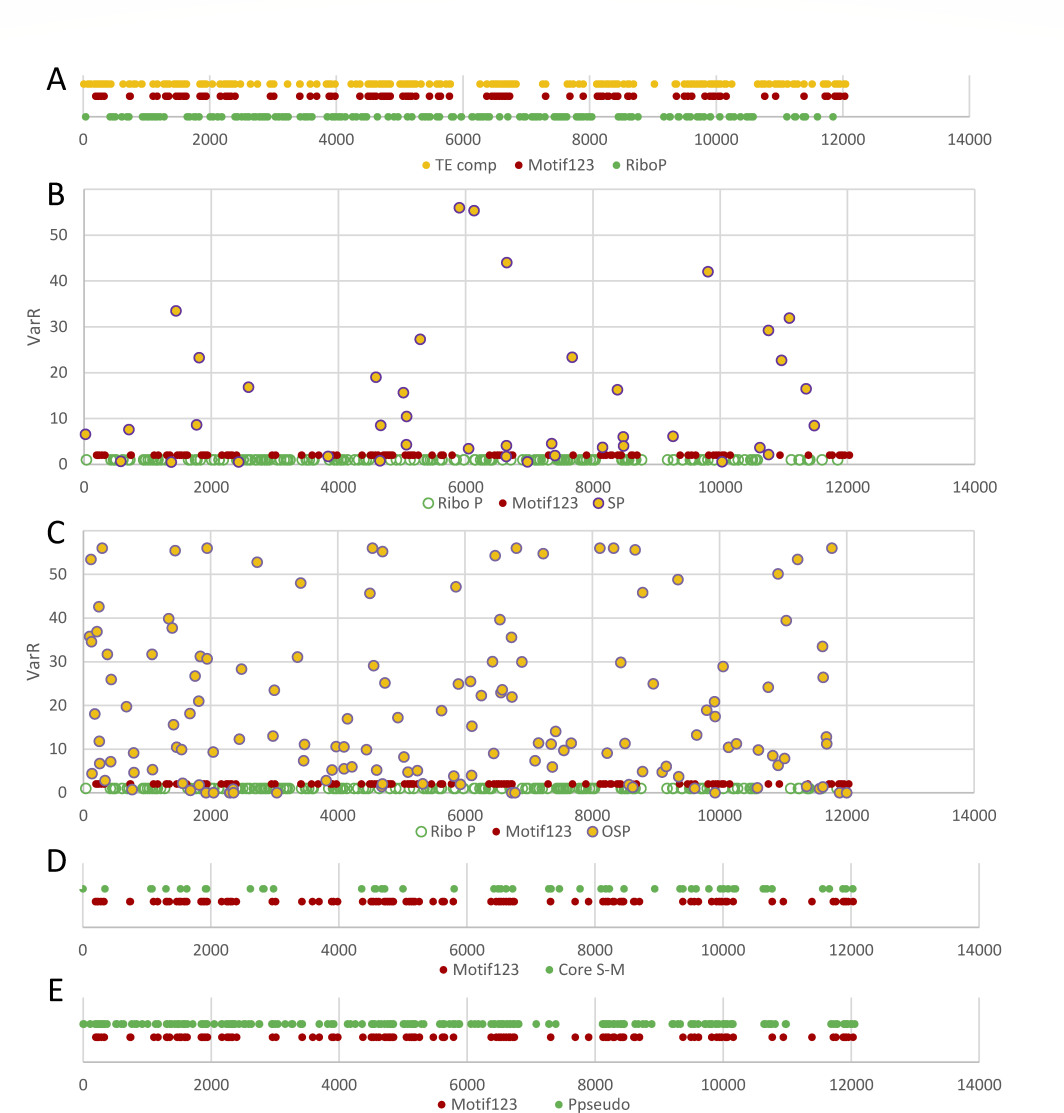
Position of TEs and genes in the DNA in relation to the three MoIsw2 palindromic DNA binding motifs (Motif123) (**A**) Ribosomal genes. (**B**) Genes for SP with conserved domains (generally larger secreted proteins) including VarR values. (**C**) Genes for OSP without any conserved domains identified (generally small secreted proteins and peptides) also included VarR values. (**D**) Core secondary metabolite (Core S-M) genes. (**E**) Potential pseudogenes (Ppseudo) not expressed in any treatment.

For typical housekeeping genes, like the genes encoding ribosomal proteins (44), their expressions depend on the overall rate of translation and should be quite constant between experiments. These genes are mainly found further away from the MoIsw2 binding sites and TEs, confirming that the ribosomal proteins are in regions with more stable expression always accessible for TFs (Fig. 6A). On the other hand, well-expressed genes that are important for interaction with the environment, such as secreted proteins with identified domains (SP) and smaller other secreted proteins (OSP) (53) are positioned in the more variable genomic regions closer to the MoIsw2 binding motifs (Fig. 6B and C).

Other genes that are important in interaction with the environment are secondary metabolites. We used the AntiSmash web server and submitted the whole *M. oryzae* DNA sequence downloaded from the Broad Institute database to get an extensive list of putative core secondary metabolite genes. We found 64 core secondary metabolite genes and plotted their positions along the ordered gene positions (Fig. 6D; Supplemental data 8, downloadable through Table S2) and found that they are mainly positioned close to the MoIsw2 binding motifs.

Epigenetic silencing can, if it is persistent from generation to generation, cause the silenced genes to accumulate deleterious mutations since the genes are never expressed and needed in the ecological niche the organism lives. We consider genes not expressed in any of the downloaded RNAsec data sets as potential pseudogenes. Since the misuse of the pseudogene term does not make sense, we follow the nomenclature earlier suggested (63) and only use the term pseudogene alone for sequences that look like genes and are not expressed at all under natural conditions. Consequently, due to limited knowledge of most microorganisms’ natural lifecycles, it is difficult to definitively classify a gene as a vestigial gene or a pseudogene. However, if a gene is rarely expressed under natural evolutionary relevant circumstances and accumulates mutations over time rendering it non-functional, it may eventually become a pseudogene. Several genes close to the *MoISW2* binding sites were not expressed at all in any of the 54 downloaded RNAseq data sets (Fig. 6E; Supplemental data 4, downloadable through Table S2), indicating that these genes could be or become vestigial genes or pseudogenes without biological roles. Thus we call them potential pseudogenes following the concept of pseudogene recently suggested (63). Our observation that these genes are or have become potential pseudogenes during relatively recent evolution is exciting since some of these genes close to the MoIsw2 binding sites are annotated as avirulence effector genes important for rice cultivar resistance to different strains of *M. oryzae* in the gene-for-gene hypothesis (see Table 1).

### DNA binding genes

As many as 65 genes closest to the MoIsw2 binding sites were classified as DNA-binding and were among the significantly enriched genes upregulated in Δ*Moisw2* (Fig. 4B; Supplemental data 13, downloadable through Table S2). These genes could be encoding conventional TFs, suppressors, or other DNA binding proteins needed when interacting with the biotic and abiotic environment potentially of high importance during pathogenesis.

The 12 DNA-binding genes most suppressed by MoIsw2 activity (upregulated in the mutant) are mainly involved in DNA repair essential for DNA synthesis and growth. Two TFs and several helicases, as well as other genes needed for DNA repair during and after DNA synthesis, are among the most regulated genes due to the deletion. A fungal-specific gene involved in (sexual) sporulation is also in this list of 12 DNA-binding genes most suppressed by the MoIsw2 activity (Table 2).

**TABLE 2:** Annotation of the 65 DNA binding genes identified in the analysis shown in Fig. 3C^*a*^

Gene	Annotation
MGG_02762	MGG_02762. ATP-dependent RNA helicase DED1; belongs to the DEAD box helicase family
MGG_06470	MGG_06470, DNA repair helicase RAD25 (835 aa)
MGG_05948	MGG_05948 zinc knuckle domain-containing protein. This domain is a zinc-binding domain of the form CxxCxxxGHxxxxC from various species. It is found in the MPE1 protein from *Saccharomyces cerevisiae* which is a component of the cleavage and polyadenylation factor complex important for polyadenylation-dependent pre-mRNA 3'-end formation
MGG_01990	**b-ZIP transcription factor IDI-4 (induces autophagic cell death in the fungus *Podospora*)**
MGG_00868	Global transactivator; superfamily II DNA or RNA helicase, SNF2 family (transcription, replication, recombination, and repair)
MGG_02429	KOG4062 6-O-methylguanine-DNA methyltransferase MGMT/MGT1, involved in DNA repair replication, recombination, and repair
MGG_07015	DNA repair protein Rad7
MGG_11518	G/U mismatch-specific uracil DNA glycosylase
MGG_04428	**Zinc finger transcription factor ace1**
MGG_07118	Sporulation-specific protein 5 (needed for sexual spore formation?)
MGG_05995	MGG_05995, *Magnaporthe oryzae* 70-15 hypothetical protein (244 aa), translin family protein, Translin family (PF01997). If translin, a review about that is in reference 64
MGG_04429	ATP-dependent DNA helicase MPH1; ATP-dependent DNA helicase is involved in DNA damage repair by homologous recombination and genome maintenance

^*a*^ These 12 genes are the most upregulated in *DMoISW2* compared to the expression in the background Ku80-strain. Annotation from NCBI. Note: these are the genes that are most repressed by the MoIsw2 activity. The bold genes are TFs.

### Functional classification of genes potentially affected by the MoIsw2 activity

We have shown above that genes under *MoISW2* control that are more expressed in the background *Ku80* strain compared to the Δ*Moisw2* strain are enriched for gene classes associated with secondary metabolism as well as biomass growth (anabolism) (Fig. 4B), while the genes that are downregulated in the mutant are connected to aerobic metabolism and stress (Fig. 4C).

Using the *MoISW2* ChIP-seq data, we investigated the FunCat classification of all genes adjacent to or in ChIP sequence hits to find out what types of genes are overrepresented and which are depleted. First, we removed double ChIP peak sequence hits close to the same gene so as not to count the same gene twice. We then investigated the list of genes that are closest to the identified MoIsw2 binding sites. These genes should be targeted by MoIsw2 and might be under positive or negative control from MoIsw2. According to our previous analysis (above), they should not belong to growth (anabolism) but be genes active when oxygen is consumed, and the substrates oxidized (catabolism) (42, 65). In addition, the binding sequences are located close to avirulence genes and retrotransposons, so their regulation can shift depending on retrotransposon transpositions. Note that since ChIP-seq only shows potential binding to DNA *in vitro,* we cannot say if these genes are up or downregulated; only that a regulation influenced by MoIsw2 is possible.

**Fig 7:**
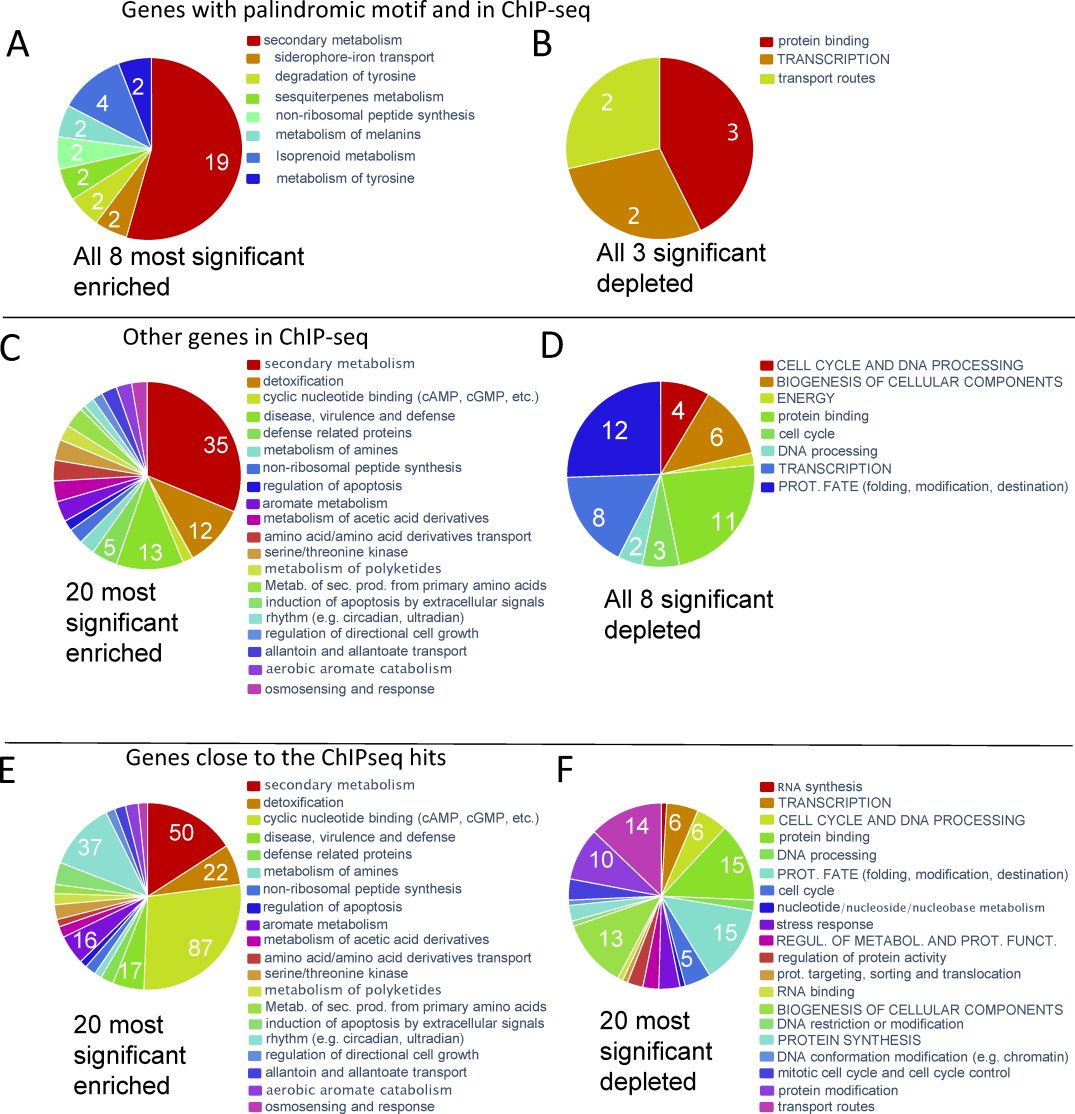
FunCat classification of genes at or close to the MoIsw2 DNA binding sites. (A and B) Significantly enriched and depleted classes of genes in the MoIsw2 ChIP-seq binding assay that also has the MoIsw2 DNA-binding palindromic motif in their upstream region. (C and D) Significantly enriched and depleted classes of all genes with ChIP-seq sequences with MoIsw2 binding to their upstream regulatory DNA sequences. (E and F) Significantly enriched and depleted classes of genes closest to intergenic hits for MoIsw2 binding, excluding the palindromic motif hits.

Genes with ChIP binding hits closest to the MoIsw2 palindromic DNA binding motif site are mainly enriched for genes involved in secondary metabolism and other gene classes important for biotic interactions (Fig. 7A). In contrast, categories of genes for biomass growth and housekeeping (anabolism) are depleted (Fig. 7B).

Many other genes within ChIP-seq sequences could be affected since they are found in the ChIP sequence. These genes are many more than those closest to the MoIsw2 palindromic DNA motif2 sites. For these genes (Fig. 7C), we find more gene classes including detoxification and signaling (cyclic nucleotide-binding), disease, and defense, and these are all important gene classes involved in biotic and abiotic interactions with the environments. Depleted are again gene classes characteristic for anabolism (biomass growth) (Fig. 7D).

Finally, the genes in the ChIP-seq data but not with the most common motif hits (not containing motif 2 sites) are enriched for secondary genes, and genes involved in abiotic and biotic interactions (Fig. 7E). Genes needed for biomass growth are depleted (Fig. 7F).

### Functional classification of 500 genes showing the highest VarR and DelR values

**Fig 8:**
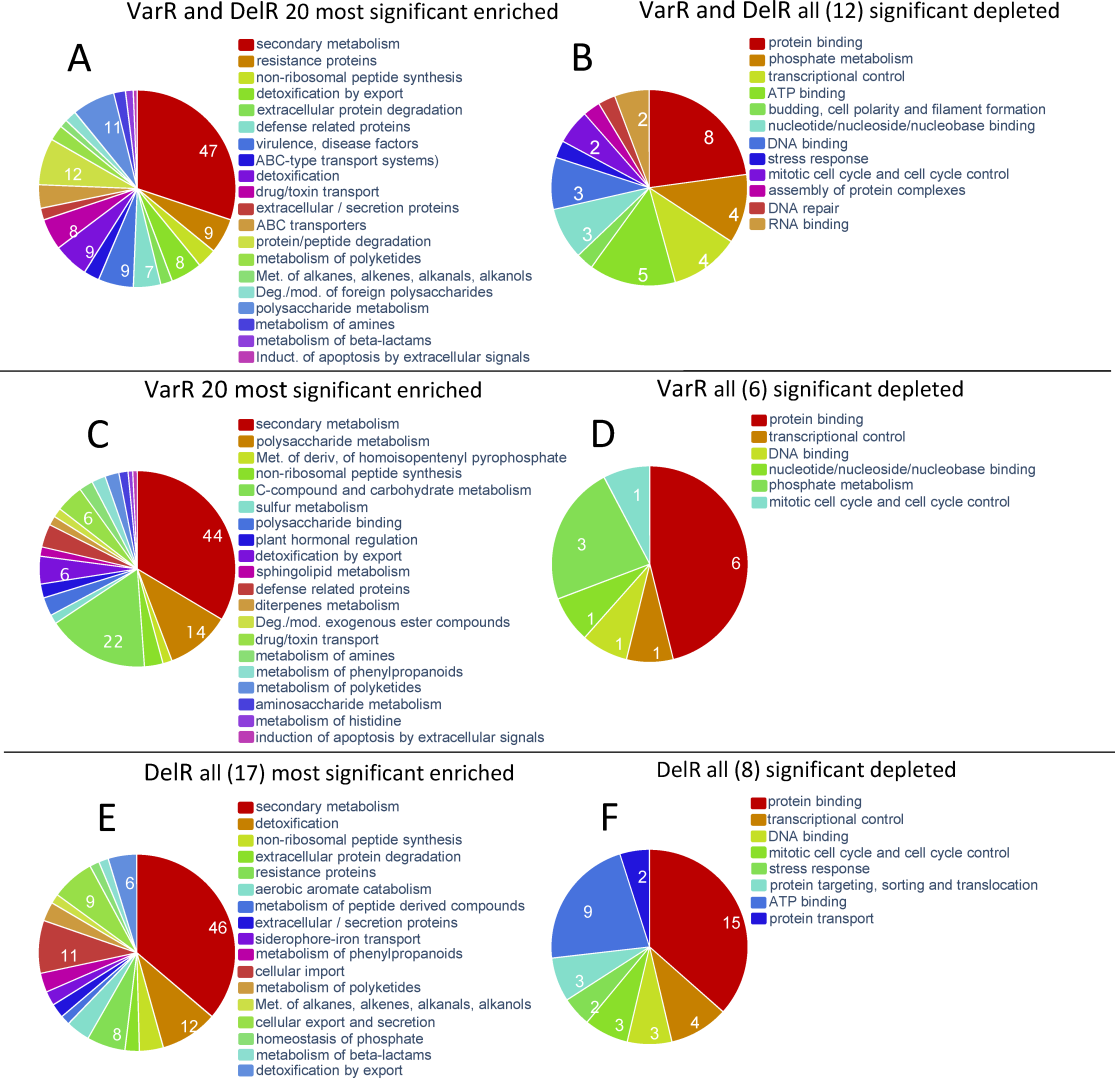
FunCat classification of 500 genes with the most variable gene expression between experiments. (**A**) VarR and DelR enriched. (**B**) VarR and DelR depleted. (**C**) VarR alone enriched. (**D**) VarR alone depleted. (**E**) DelR alone enriched. (**F**) DelR alone depleted.

As we have seen above (Fig. 5 and 6) the genes in the vicinity of MoIsw2 binding motif 123 in the downloaded RNAsec plant infection experiments are generally located in more variable expressed DNA regions (VarR). In these regions, most TEs, secreted proteins, and core secondary metabolite genes are co-located. To obtain more information about regulated functional gene classes we again used FungiFun and performed a functional classification of the 500 most variable genes (VarR) in the downloaded data from plant infection experiments and compared these with the genes most affected by the *MoISW2* deletion (DelR) (Fig. 8; Supplemental data 12, downloadable through Table S2). Few of these genes are likely to be directly regulated by MoIsw2, but access to them for gene regulation is likely affected. In addition, similarities and differences between VarR and DelR should indicate regulations biased of genes important for plant interactions or plate growth, respectively.

Genes categories required for coping with the abiotic and biotic environment are enriched among the 500 with the highest VarR and DelR values, suggesting that similar gene functions are in principle affected by MoIsw2 activities (Fig. 8A). The depleted functional gene classes are growth-related genes (anabolic) and some stress-related genes (Fig. 8B).

Genes categories that are needed to cope with the abiotic and biotic environment are again enriched among the VarR or DelR 500 most differentially regulated gene classes (Fig. 8C and E). Depleted are again growth-related genes (anabolic) and stress-related gene classes (Fig. 8D and F).

The fungal biomass grown *in vitro* had recently been transferred from a mostly stationary state (agar plugs) to a liquid medium for 48 h inducing strong growth. Therefore, variations in the genes affected by the deletion (DelR) are expected for genes responsible for how quickly the biomass responds to the new situation of nutrient abundance even in genes not affected by MoIsw2 activities. (Fig. 8E and F). But in general, the functional gene classes among the high VarR alone and the high DelR alone gene classes, respectively, are similar.

**Fig 9:**
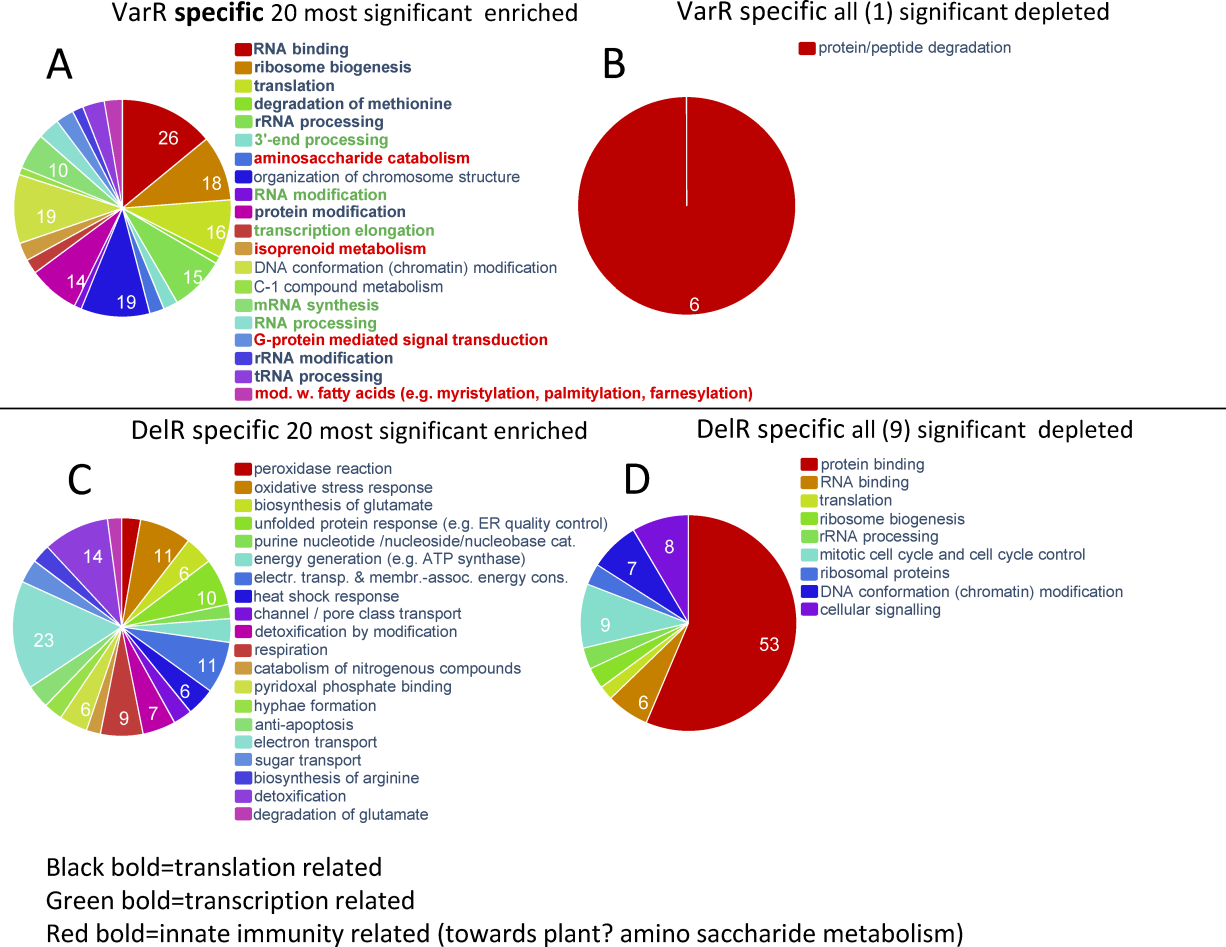
Differences between 500 VarR-specific and DelR-specific functional gene classes illustrate the difference in conditions between *in planta* experiments and *in vitro* experiments. (**A**) VarR-specific significantly enriched functional gene classes specifically responding *in planta*. Color-marked texts are the functional gene classes of special interest for plant pathogenicity (see text). (**B**) VarR-specific significantly depleted functional gene classes specifically responding *in planta*. (**C**) DelR-specific significantly enriched functional gene classes specifically responding *in vitro.* (**D**) DelR-specific significantly depleted functional gene classes specifically responding *in vitro*.

Gene classes specifically enriched in the VarR (VarR-specific genes) are expected to be gene classes responding to conditions of fungal-innate immunity (66) (reacting to plants in this case) (Fig. 9A and B). Because of the increased nutrient availability in the liquid medium used for producing the biomass, the DelR-specifically enriched and depleted gene classes (Fig. 9C and D) should indicate gene classes important to regulate during fast aerobic growth, hyphal biomass increases and nutrient uptake from the environment *in vitro*.

## DISCUSSION

### Deletion effects on phenotypes, and regulation (DelR) compared with variability in regulation between RNAseq experiments (VarR)

When *MoISW2* is deleted, the general *in vitro* growth of the fungus (Fig. 1) was negatively affected, as was the sensitivity to SDS and NaCl. Conidiation was completely abolished as was pathogenicity, and the MoIsw2-GFP fusion accumulated in the nucleus as expected for a nuclear-localized protein. The strong effect on infection and the fact that Isw2 proteins are known to influence the regulation of many genes in genomes by local chromosome remodeling around its several DNA binding sites (28) suggests that *MoISW2* may be a “master regulator” for regulating the many fungal defenses against plant defenses when inside the plant. This is because the latter and virulence factors (e.g., secondary metabolism) are particularly upregulated during the transition from biotrophy to necrotrophy, and in the necrotrophic stage (67, 68) when membrane effects on pathogens are particularly pronounced and the infecting fungus has to contend with plant ROS defense mechanisms.

We found that potential effector genes such as avirulence genes, secreted proteins, and core secondary metabolite genes are located in the DNA regions that appear to be under the control of MoIsw2. The protein appears to have the role of a master regulator of TF and repressor gene to access the genes necessary for the fungal responses to the biotic and abiotic environment (Fig. 5 to 7A). These types of genes are located in regions of DNA that are differentially regulated in the Δ*Moisw2* mutant compared to the K80 background (the DelR data). These regions are also the same regions with the highest variability between experiments in a set of RNAseq data from the 55 downloaded RNAseq data sets of experiments *in planta* (Fig. 5) (VarR data). That is what one might expect for niche-determining genes that do not have much to do with basic metabolism.

The overall regulatory effect of all genes in the two datasets (VarR and DelR) were correlated, as they should be if the regulated regions in the two data sets were the same and strain-specific and not experimental, and dependent only on the organization of chromatin by MoIsw2 in the particular strain. As an extra control for this discussion, we investigated if the two measures of MoIsw2 influence on gene variation are correlated with the gene distance from the MoIsw2 DNA binding locations in the ChIPseq experiment (Fig. 5) and not just correlated in expression for all genes (Fig. S8). Both VarR and DelR show a similar exponential decrease in values with the distance from the MoIsw2 binding locations (Fig. S9).

Growth in a plant is stressful for a plant pathogen. The fungus must compete with the plant for all nutrients during the biotrophic stage without revealing its existence so that the plant’s immunity responses do not start before entering the necrotrophic stage (67, 69, 70). During the transition to the necrotrophic stage, the pathogenic fungus must overcome both plant defenses and nutrient limitations, as nutrient limitations may occur (71). As evidence for a strain-specific “master regulator” function of MoIsw2 regulating responses to environmental conditions, we find that many functional gene classes related to fungal defense and virulence are regulated and positioned in the variably regulated chromosomal regions that appear to be under the control of MoIsw2 activity (Fig. 8 and 9). In addition, there is a difference in the regulation of avirulence genes between the *M. oryzae* strains, and these genes are located near the MoIsw2 binding sites in the DNA, further supporting the strain specificity of MoIsw2 as a master regulator (Table 1).

### Regulation of chromatin condensation by nucleosome positioning and MoIsw2 is a true Isw2

Direct interactions with His4 could not be detected using a yeast two-hybrid assay. The yeast interaction of Isw1 and Isw2 with His4 are transient (about 200 and 80 bindings per minute, respectively) and require a continuous supply of ATP (48). Similarly, the interaction has been measured in human cell lines *in vivo* using advanced microscopy and spectroscopy techniques and found to be transient (10–150 ms) (34, 35). It is therefore not surprising that our attempts to detect an interaction between MoIsw2 and MoHis4 using the yeast two-hybrid method were in vain. On the other hand, the expression of MoIsw2 with the genes Isw2 proteins is known to physically interact with and form a functional Isw2 protein complex (33) and is well correlated (Fig. 2A through F). In addition, *MoISW2* could complement yeast *Scisw2* (Fig. 2G) suggesting that MoIsw2 is a true Isw2 with a conserved Isw2 protein function. Further support is that it has a conserved order of domains (Fig. 1A; Fig. S3) and the entire protein is well conserved phylogenetically within fungi-metazoa-protozoa (Fig. S1 to S3).

### Gene regulation by MoIsw2 activity

*In vitro* Isw1 translocates nucleosomes away from other nucleosomes, while Isw2 translocates toward the center of DNA fragments and other nucleosomes *in vitro* when nucleosomes are bound to relatively short DNA pieces (27, 72). The same occurs *in vivo*, where Isw2 was found to be primarily involved in the targeted regulation of DNA access near the Isw2 DNA binding site (28), as we also established (Fig. 4 and 5; Table 1).

The Isw2 activity is ATP-dependent (30–32). Thus, the regulation of genes adjacent to the DNA-binding site is likely to be highly dependent on ATP availability and in metabolic competition for ATP in the cytoplasm-nucleus compartment. In eukaryotes, the rate of ATP generation by fermentation is much faster but less efficient than oxidative phosphorylation when sufficient substrate is available for rapid fermentation. In yeast, aerobic growth is for example characterized by periodic phases of DNA replication without mitochondrial oxidative activity (aerobic glycolysis), probably to protect DNA from mutations in growth phases of oxidative phosphorylation and interaction with the environment (nutrient uptake) (65). Thus, in *M. oryzae,* MoIsw2 may be an ATP-regulated switch (42) between a state of rapid ATP formation during aerobic glycolysis accompanied by DNA synthesis and biomass growth together with DNA quality control, and a more nutrient-efficient state of oxidative phosphorylation growth with high ATP yield at a slower rate, active oxidative defenses, and interaction with the abiotic and biotic environment that constitutes the ecological niche of *M. oryzae*. In support of this, we found that genes upregulated in the *ΔMoisw2* mutant and thus repressed by MoIsw2 under other, more ATP-limited aerobic growth conditions are involved in rapid growth and DNA synthesis, as well as DNA quality control. The genes downregulated genes in the mutant were those involved in oxidative phosphorylation, stress management, and biosynthesis of secondary metabolites (Fig. 4B and C; Table 2).

### Regulation by MoIsw2 is dependent on distance from the MoIsw2 DNA binding sites

The variation in gene regulation in the Δ*Moisw2* mutant strain compared to the *Ku80* background strain (DelR) and the variation between RNAseq experiments in different labs (VarR) are dependent on the distance to the MoIsw2 DNA binding motif sites (Fig. 5; Fig. S9). This is consistent with the observation that regulation is a consequence of the Isw2 positioning of the closest nucleosomes, giving more sliding space to surrounding nucleosomes (33), and consequently increasing DNA access for TFs and repressors positioned slightly further away. Genes affected by MoIsw2 activities that are physically close to its palindromic binding sites in the DNA include, for example secreted protein and core secondary metabolite genes, whereas typical housekeeping genes like genes encoding ribosomal proteins are much further away (Fig. 6). In support of this, we found that the 500 most differentially regulated genes (high VarR and DelR) with most variable gene expression were significantly enriched for functional gene classes needed to respond to the biotic and abiotic environment and significantly depleted where typical housekeeping genes (Fig. 8). Interestingly, and consistent with this interpretation, the 500 genes specifically upregulated in VarR (during plant pathogenesis) and in DelR (growth *in vitro*) are genes that reflect the two different biotic/abiotic environments for the fungus (Fig. 9). The functional gene classes that are particularly enriched in VarR (especially the ones in red text in Fig. 9) are expected to be involved in fungal innate immunity (66) and protection against plant innate immunity such as the metabolic removal of fungal cell wall derived amino saccharides (chitin oligomers) (Fig. 9A) that is a well-studied trigger of plant immunity (73).

### Conserved and variable DNA

Our observations suggest that the *M. oryzae* genome is organized into regions with constant expression, and variable expression depending on the environment. The regions with variable expression are close to MoIsw2 DNA binding sites and contain genes important for the interaction with the environment (during biotic and abiotic stresses), while regions with less variable expression contain housekeeping genes. Such a division of the fungal genome into regions of housekeeping genes and niche-determinant faster-evolving genes is consistent with previous findings in the relatively closely related *Fusarium graminearum* (74), and also in *V. dahliae* (38).

Palindromic sequences are characteristic of retrotransposons, which are mainly found near stress-related genes and avirulence genes in the genomes of fungal plant pathogens. Fungal avirulence genes are plant-pathogen effectors that trigger different plant immunity responses depending on the plant variety and form the basis for the crop-specific resistance of plants to certain pathogen strains (the gene-for-gene relationship) (55) and we find them in the vicinity of the palindromic MoIsw2 binding motifs accompanied by TEs (Table S2; Fig. 6A). Most of the MoIsw2 DNA binding sequences (the ChIPseq sequences) (90%) contained previously identified TEs (53) (Fig. 3). Palindromic DNA motifs confers genetic instability to the genome at the site of the motifs (75, 76) that are linked to TEs. Stress, virulence, and stress-related genes are also associated with transposable elements in both *V. dahliae* (38) and *M. oryzae* (40) and we see this in our study (Fig. 6 to 9).

Mutation bias was recently described as a new concept for the plant *Arabidopsis thaliana* (45). Genes with higher mutation rates are typically involved in biotic and abiotic interactions. The gene with the highest mutation rate was a gene that can degrade chitin to oligomers, which is present in symbiotic (pathogenic and non-pathogenic) fungi and insects. The oligomers work as a microbe associated molecular pattern stimulating plant defenses (45).

Transposable element activity appears to play an important role in fungal speciation (38) and tends to accumulate in genomes and expand the genome with repetitive sequences (56, 57) unless there is some evolutionary constraint against such expansion (56, 77). The availability of phosphorous is a possible constraint (78) since a considerable part of the cell phosphorous is bound up in DNA and not available for other purposes (79). Fungal plant parasites can obtain phosphorous directly from their plant hosts by degrading plant tissue and are not constrained by limited resources as fungi in the soil are (78). Thus, one would expect that plant parasitic fungi have more retrotransposons to adapt more quickly to changes in plant resistance (the gene-for-gene hypothesis) and therefore have relatively large genomes without necessarily more active genes. This is exactly what they have (56, 57). Endophytic fungi, which “mainly” have to coexist with their host in their life cycle and therefore have to compete with it for phosphorous, should therefore have smaller genomes than pathogens, which they also seem to have (56).

Our results show changes in the occurrence and regulation of avirulence genes between two *M. oryzae* strains with different pathogenicity for different rice cultivars (Table 1). In addition, we did not detect expression for some putative avirulence genes near the MoIsw2 DNA binding sites suggesting that these genes could be potential pseudogenes in these strains. This could indicate retrotransposon-assisted genetic evolution of inherited epigenetic changes for regulation in response to the environment (resistance of the plant cultivar), as found for *V. dahliae* (38). Such regulation can become genetically fixed, first by loss of potential regulation (turning downregulated genes into factual pseudogenes) and much later by complete loss of the gene or alteration of the potential pseudogenes so that it cannot easily be recognized as a gene. Isw2 regulation is thus a mechanism that could result in the described biased faster evolution of genes (39). Most of the potential pseudogenes in the downloaded data sets from many laboratories and not expressed in any of them were close to the MoIsw2 DNA binding sites with palindromic motifs (Fig. 6E) supporting the hypothesis of a mechanism involving MoIsw2 activity in the creation of pseudogenes, and subsequent complete elimination of genes by rendering them unrecognizable as genes through accumulated mutations. A prerequisite for this is that the ecological niche the plant constitutes does not change rapidly. If it does that, through spontaneous development of plant resistance or breeding, such changes could easily reactivate the silenced avirulence genes by stress-induced TE repositioning of the genes to chromosomal regions and alter MoIsw2 activity and the plant resistance would be broken.

Our results further indicate that MoIsw2 regulates a switch between rapid aerobic glycolysis accompanied by fast DNA synthesis, and slower but nutrient-efficient aerobic growth with less DNA synthesis and coping with abiotic and biotic stress, or in other words, niche fitness. Loss of Isw2 function in a unicellular eukaryote, or the early stages of filamentous fungal growth from a spore renders the fungus unable to to survive in a natural aerobic environment as it becomes dependent on aerobic fermentation for ATP synthesis, which is not substrate efficient although it generates ATP at a high rate albeit inefficiently (80–82). However, restricting eukaryotic cells to aerobic fermentation generally leads to uncontrolled cell growth and oncogenesis of cancer cells in a long-lived multicellular organism (the so-called Warburg effect) (83–86), as ATP and fermentable nutrients can be received from surrounding cells and other tissues via interstitial fluids and the blood circulation. Thus, mutations in the *SMARKA2* or *SMARKA4* genes that encode Brm and Brg1 proteins that are orthologues Swi2/Snf2 (ISW2) in fungi can cause cancer and make cancer grow fast and become malignant (87–89).

On the other hand, a general switch to aerobic glycolysis and a rapid upregulation (within a few hours) of innate immunity-related genes and their ATP-dependent translation is positive and required for inflammatory responses of tissues to pathogens but need to be balanced and could overreact causing sepsis if these responses become systemic (90). A fast upregulated *de novo* protein synthesis is characteristic of innate immunity defenses against microbes needing rapid translational responses and is general for eukaryotes have been shown for the fungus *F. graminearum* (66). Fast upregulated plant innate immune responses can be inhibited by fungal-derived immune system inhibitors like fungal trichothecenes that inhibit cell translation and are upregulated in plant infection (91). These compounds have most likely evolved to attenuate plants‘ innate immunity responses toward fungi. In line with a fungal innate immunity scenario (66) with the need for fast upregulation of fungal defense proteins, genes involved in RNA synthesis and RNA processing were found to be upregulated in the Δ*Moisw2* mutant (Fig. 4B).

### MoIsw2 DNA-binding and activities are a possible cause of mutation bias by adapting the fungus to new challenges

*MoISW2* is likely an important player in a mechanism that supports both mutation bias and adaptation to new circumstances. The combined activities of TEs and MoIsw2 will result in a two-speed evolution of the fungal genome (38)—a slow, mostly random evolution and a fast, adaptation-directed one. The faster rate of evolution can be called natural adaptation-directed fast evolution (NADFE), a new concept in line with artificial adaptation-directed fast evolution that can be performed in a laboratory (92). As transposon DNA containing MoIsw2 palindromic DNA binding sites seems necessary for MoIsw2 DNA binding and activity the proposed mechanism is as follows: (i) stress-activated transposon activity, which is known to generate genomic instability, together with MoIsw2 activity creates a new adaptive regulatory landscape (39). That epigenetic landscape can be created and stabilized relatively quickly and lead to mutation bias (45). Due to the mutation bias, the stabilized expression landscape can become further stabilized and fixed by subsequent mutations. The whole process could be the mechanism sought for a Lamarckian-Darwinian synthesis that can explain the fast evolution of new traits that require many combined mutations simultaneously to become positive. Many simultaneous mutations cannot be reconciled with strict random evolution combined with sexual recombination and natural selection as the cause of the observed direction and the needed speed of evolution of specific traits when new niches become available, or drastic niche changes occur (93). The chance of multiple mutations that only together can become positive is theoretically much more likely with NADFE.

### Perspectives for future research

#### The evolution of plant-pathogen interaction

The effect of exposure of *M. oryzae* strains to different MoIsw2 activities and retrotransposon activities on the rate of genetic adaptation to a change of rice cultivar is a potential future topic that could be used to understand how the pathogen adapts to different resistant cultivars and break plant resistance.

#### Agrochemicals

Searching for potential agrochemicals to manipulate MoIsw2 activities on His4- and DNA-binding is tempting since *MoISW2* is crucial for plant pathogenicity. The close resemblance of MoIsw2 to mammalian proteins with similar functions indicates that such chemicals are likely negatively affecting orthologue proteins in mammals and might be carcinogenic to humans since even small mutations in *ISW2* orthologues in mammals can cause cancer (87, 88).

#### Histone 4

We noted 2 *MoHIS4* genes with significant differences in DNA sequences (gene duplication many generations ago). These genes encode for identical proteins, but they are regulated in distinct ways, and it appears that *M. oryzae* needs both genes (Fig. 2F). This fact is of interest since His4 proteins are the nucleosomal proteins MoIsw2 interact with (48), and *HIS4*s are known to exhibit the highest degree of purifying evolution (94).

#### Gene expression variability

The most interesting genes for biotic and abiotic interactions seem to be the genes with the highest variability between experiments (VarR) and that creates problems when investigating the role of these genes through deletion studies. The common practice of only using three replicates of the WT and the deletion strains for measuring the effects of the deletions is probably not enough to detect changes compared to the background even when the phenotype is recorded as a change in gene regulation of a reporter gene. We need to increase the number of replicates to five or more, especially in the wild-type background. If that is done, we can either statistically prove the type of distribution, or use non-parametric methods so as not to assume a normal distribution, or more correctly in most cases a lognormal distribution, of the expression.

### Conclusion and consequences of MoIsw2 functions that are positive for the fungus’s ability to stay pathogenic

- The MoIsw2 functions will result in adaptive silencing of virulence/avirulence genes without gene loss due to mutations of the encoding DNA. In other words, virulence genes are not quickly lost from the adapted strains but can be “called upon again” if the available hosts reasonably fast change their resistance in the classical gene-for-gene scenario (95).
- Adaptive silencing of virulence genes is often found close to transposable elements (96). Such transpositions will lead to biased fast gene evolution (45) of especially genes involved in biotic interaction and further support the gene-for-gene hypothesis. Thus the Isw2-TE activity is a possible mechanism behind the speedy appearance of new virulent strains of pathogens capable of attacking newly developed resistant plant varieties (96, 97).

## MATERIALS AND METHODS

### Fungal strains and media

*M. oryzae* B. Couch anamorph of the teleomorph *Pyricularia oryzae* Cavara was used for this research. As background strain, we used *Ku80* (generated from the WT strain Guy11) to minimize random integration events when transformed (98). The susceptible Indica rice (cv. CO-39) and barley (cv. Golden Promise) used for the fungal pathogenicity tests were from the seed bank of our laboratory. For both ChIP-seq and RNAseq, the strains used were grown and harvested similarly.

### Knockouts, complementation, and verifications

The *MoISW2* is a *MYB* gene and *MYB* gene deletion vectors were constructed in the plasmid pBS-HYG by inserting 1 kb up- and downstream fragments of the target gene’s coding region as flanking regions of the HPH (hygromycin phosphotransferase) gene (99). No less than 2 µg of the deletion vector DNA of the target gene was introduced to *Ku80* protoplasts, and transformants were selected for hygromycin resistance to perform gene deletion transformations. Southern blotting was conducted to confirm the correct deletion using the digoxigenin (DIG) high prime DNA labeling and detection starter Kit I (11745832910, Roche Germany). The *MYB* gene complementation vectors were constructed by cloning the entire length of the target gene with the native promoter region (about 1.5 kb) to the pCB1532 plasmid. When making the complementation vector, GFP was linked to the C-terminal of the target genes to study the sub-cellar localization of Myb proteins. The constructed vector DNA was introduced into the mutation protoplast for gene complementation, and resulting transformants were screened using 50 µg/mL chlorimuron-ethyl to select successful complementated strains. Detailed fungal protoplast preparation and transformation methods have been described previously (99). All primers needed for the knockout and complementation are listed in Table S1. The sub-cellar localization of Myb proteins was observed using a Nikon A1 confocal microscope (Nikon, Japan). The GFP and RFP excitation wavelengths used were 488 nm and 561 nm, respectively.

### Heterologous complementation of yeast ISW2 phenotype

*S. cerevisiae* wild-type strains BY4741 and ISW2 mutant Δ*scisw2* were obtained from Professor Chen Ding and Dr. Yang Meng at Northeastern University, China. The constructs containing full-length *MoISW2* were cloned into the pYES2 vector and then transformed into Δ*scisw2* to obtain Δ*scisw2/MoISW2*. All strains were grown in yeast extract peptone dextrose (YPD) growth medium (1% [wt/vol] yeast extract, 2% [wt/vol] peptone, and 2% [wt/vol] glucose) with or without 100 µg/mL 5-FC.

### Colony growth and infection phenotype measurements

Vegetative growth was tested by measuring the colony diameter of the strains after 10 days of growth on CM at 25°C under 12 h-to-12 h light and dark periods. Conidia production was evaluated by flooding the 12-day-old colonies with double distilled water, filtering out the mycelia with gauze, and counting the conidia using a hemacytometer. The conidiophore induction assay was performed by excising one thin agar block from the fungal colony and then incubating it in a sealed chamber for 24 h with constant light (100). Mycelia appressoria was induced by placing a suspension of mycelial fragments on a hydrophobic surface in a humid environment at 25°C for 24 h. The pathogenicity assay on rice and barley was performed by inoculating intact or wounded plant leaves using agar disks (101). The inoculated plants were kept in a sealed chamber with a 90% relative humidity at 25°C for 24 h before the inoculated plants were removed from the chamber to allow disease symptoms to develop for 4–5 days. The pathogenicity assay on excised barley and rice leaves was performed by cutting a small block from the agar culture of the fungus and placing it on excised leaves for 5 days in a moist chamber for disease development (102). Sexual reproduction was tested by crossing the tested strain with the sexually compatible strain TH3 on OM plates and then incubating at 19°C for 30 days with continuous light. The perithecia and clavate asci were photographed in a microscope equipped with a camera (OLYMPUS BX51).

### Biomass production for ChIPseq and RNAseq

For ChIP-seq, the complemented strain, MoIsw2-GFP, and the strain *Ku80* were used, and for RNAseq Δ*Moisw2* and *Ku80* were used. The strains were grown on Complete medium 2 (CM2) (all are quantities L^−^): 20× mineral salts solution 50 mL, 1,000× trace element solution 1 mL, 1,000× vitamin solution 1 mL, D-glucose 10 g, peptone 2 g, casamino acid 1 g, yeast extract 1 g, pH 6.5. For agar medium add 15 g agar. Supplementation with 20× mineral salts solution was as follows (per 1,000 mL): NaNO_3_ 120 g, KCl 10.4 g, MgSO_4_·7H_2_O 10.4 g, KH_2_PO_4_ 30.4 g, and with 1,000× vitamin solution (per 100 mL): biotin 0.01 g, pyridoxin 0.01 g, thiamine 0.01 g, riboflavin 0.01 g, PABA (p-aminobenzoic acid) 0.01 g, nicotinic acid 0.01 g, and 1,000× trace element (per 100 mL): ZnSO_4_·7H_2_O 2.2 g, H_3_BO_3_ 1.1 g, MnCl_2_·4H_2_O 0.5 g, FeSO_4_·7H_2_O 0.5 g, CoCl_2_·6H_2_O 0.17 g, CuSO_4_·5H_2_O, Na_2_MoO_4_·5H_2_O 0.15 g, EDTA·4Na 5 g. The plates were incubated for 4–5 days at 28°C with alternating light-dark cycles (12/12). Mycelial disks (15–20) were punched out at the colony’s edge using a 5 mm diameter cork puncher. The disks were transferred to 100 mL CM2 liquid medium and shake cultured for another 48 h (160 RPM, constant temperature culture oscillator, ZHWY-2102C, Shanghai Zhicheng Analytical Instrument Manufacturing Co., Ltd.). The obtained biomass was filtered using Miracloth, frozen in liquid nitrogen, and sent on dry ice to the company performing the RNAseq.

### ChIP-seq and RNA-seq

These two techniques were carried out by the company Wuhan IGENEBOOK Biotechnology Co., Ltd., China, according to their method (Table S2, Supplemental Company Methods files 1 and 2). The last steps concerning finding motifs and enriched gene classes were carried out differently and are described in this paper. The steps not used are marked in the two files.

### Method for calculating VarR and DelR in the RNAseq data

We calculated the variability in gene expression for each gene for all the downloaded experimental data sets in the following way. First, we checked that the quality of the data was good enough. We have previously found (but not published) for our transcriptomic data that a data set is good enough to include if expressions of genes are rank-ordered and then the log-log plot of log expression level against the log-rank order should be a smooth downward slightly curved line without big gaps. Such gaps appear due to the normal RNAseq procedures calculating the expression if not enough RNA is sequenced. Then low expression values will consequently be suspect even when lots of data sets have similar low values. Also, to be able to compare different sets of data from different experiments the total expression of all genes summed should be similar or corrected to be similar (normalized) for all data sets. In that way, the expression values for each data set will be equal to a fraction of the total expression. If this is done for all data sets, we can use them all to find correlations of expression between two or more genes across many experiments and experimental conditions for the same strain of a fungus (for example time from plant inoculation in plant pathology data). In principle, it is also possible to test if these gene correlations are different for radically different conditions like *in vitro* and *in planta* or during starvation if there are lots of data sets available for the same isolate for these separate conditions.

Next, we calculated the maximum variation between experiments in the following way. The maximum expression value and the minimum expression value for each gene across the 55 transcriptomes were found. Then, we calculated the average expression for the same data. After that, we calculated the ratio of maximum expression minus minimum expression divided by the average expression to arrive at the variation in expression across all experiments for each gene (VarR). In this way any regulation up or down is treated in the same way as epigenetic-influenced access to the DNA for TFs and repressors should be treated equally.

Finally comes the problem that a few genes have their lowest expression values lower than the threshold for detecting them at all (0-values). For these data, we assumed that the expression was at the threshold value instead of 0 and set the expression to that value. In that way, we could include those data without overestimating the VarR.

The effect of the *MoISW2* mutation (DelR) was estimated in the following way to make it comparable with VarR: for each gene, we calculated the 2log ratio of the expression in the mutant divided by the expression in the background. That results in both negative and positive values. Then these values were squared to arrive at only positive values in a similar way as is done with the least square method for curve fitting to arrive at a better estimate of the deviation from the slope. There were very few genes with no detectable expression so no need for handling zero values more than excluding them. We further tested that the VarR and DelR values are correlated in two different ways (see Results and Fig. S8 and S9). The data needed for these comparisons can be found in Supplemental data 4 and 5, downloadable through Table S2.

Note that in this paper, we focus on the expression variation of all genes along the chromosomes. Thus, it is the expression landscape that is important and not the values for a single gene. In none of the methods above we used any method for excluding a gene from the analysis based on an arbitrary threshold of 2 times up or down or any other statistically significant threshold for genes. In consequence, the calculated values for a single gene cannot and should not be relied upon even if a generally increased regulation of many adjacent genes has reliability.

### Additional software and add-ins for MS Excel

#### Add-ins for MS Excel

The Fisher Exact add-in for MS Excel was downloaded from http://www.obertfamily.com/software/fisherexact.html. The Excel solver was used to fit non-standard equations to data. The solver is usually part of MS Excel but must be activated in settings.

#### Freeware

We used the freeware PAST. It is a simple-to-use but powerful freeware program available from the University of Oslo, Natural History Museum (https://www.nhm.uio.no/english/research/resources/past/) (103) version 4.08 (released November 2021). We mainly used the PAST software to do reduced major axis (RMA) regression analysis to handle errors in both the *x* and *y* variables and for regression comparisons of slopes using standard regression plots with only the *y*-axis for measured data dependent on known *x*-axis values. For simplicity, the data were handled and entered in MS Excel and then copy-pasted into PAST for analysis. The resulting plots were exported from PAST as SVG vector graphic files to be later translated into other vector graphic file formats like the one used in MS PowerPoint that can directly import SVG files so they can be edited.

### Websites used for analyses and getting necessary additional data

NCBI (https://www.ncbi.nlm.nih.gov/) was the resource for sequence downloads, blasts, and annotations, including domain annotations. BLASTP was used for BLAST comparisons of MoIsw2 and putative orthologous proteins in other organisms. COBALT was used for multiple alignments of the putative orthologous proteins. The conserved domains of some selected proteins were also found at NCBI.

The NGPPhylogeny.fr site was used to plot a phylogenetic tree using the default 1-click option.

BROAD (ftp://ftp.broadinstitute.org) was the resource to download protein sequences and gene order for the Guy11 strain (*Ku80*).

MEME is available at https://meme-suite.org/meme/ (use of MEME and FIMO) (104). See the supplemental data for MEME settings and complete results (Supplemental data 2, downloadable through Table S2). MEME motif2 was the one with the most hits and was investigated in detail. This motif2 was used in TOMTOM by using the direct link from the MEME output, for results see Supplemental data 3, downloadable through Table S2.

FungiFun2 is available at https://elbe.hki-jena.de/fungifun/fungifun.php (105). Species *Magnaporthe grisea* 70-15 contains all genes also in Guy11 (*Ku80*). Classification ontology used FunCat (contains many more plant pathology-relevant categories than Kegg or GO). For input IDs, the MGG_ codes have to be used. Advanced settings used were as follows: significance level, 0.05; significance test, Fisher’s exact test; test for enrichment or depletion; adjustment method, no adjustment; annotation type, select also indirectly annotated categories. These settings using “no adjustment” were used since the purpose was not to get super reliable enriched or depleted categories but to compare several FungiFun runs with different Gene ID sets.

### antiSMASH

The complete DNA sequence of *M. oryzae* Guy11 (*Ku80*) was downloaded from BROAD. It was then submitted to the antiSMASH (106) fungal version https://fungismash.secondarymetabolites.org/#!/start and run with default parameter settings to find the core genes predicted to be involved in producing secondary metabolites.

## SUPPLEMENTAL MATERIAL

Supplemental material mbio.01590-24-s0001.docx Figures S1-S8 and Tables S1 and S2.

ASM does not own the copyrights to Supplemental Material that may be linked to, or accessed through, an article. The authors have granted ASM a non-exclusive, world-wide license to publish the Supplemental Material files. Please contact the corresponding author directly for reuse.
