# Supplementary material for: Whole genome regulatory effect of MoISW2 and consequences for the evolution of the rice plant pathogenic fungus Magnaporthe oryzae
Source: mBio. 2024 Sep 18;15(10):e01590-24. doi: 10.1128/mbio.01590-24 (PMC11481914; doi:10.1128/mbio.01590-24)
Supplement: Supplemental material — Figures S1-S8 and Tables S1 and S2. [file mbio.01590-24-s0001.docx]

Supplemental figures

S1-S8 and Table S1-2

| Description | Seq ID | Score | Expect | Identities | Positives | Gaps | Group |
| --- | --- | --- | --- | --- | --- | --- | --- |
| ***Gaeumannomyces tritici***  **R3-111a-1** | [XP_009216200.1](https://www.ncbi.nlm.nih.gov/protein/XP_009216200.1?report=genbank&log$=protalign&blast_rank=1&RID=30DY9FYK01N)  Length: 1125 aa | 1980 bits(5129) | 0.0 | 994/1117 (89%) | 1044/1117(93%) | 6/1117(0%) | Ascomycota |
| ***Colletotrichum graminicola* M1.001** | [XP_008092352.1](https://www.ncbi.nlm.nih.gov/protein/XP_008092352.1?report=genbank&log$=protalign&blast_rank=2&RID=30DY9FYK01N)  Length: 1119 aa | 1848 bits(4786) | 0.0 | 926/1114 (83%) | 1003/1114 (90%) | 11/1114(0%) | Ascomycota |
| ***Fusarium solani*** | [XP_046134388.1](https://www.ncbi.nlm.nih.gov/protein/XP_046134388.1?report=genbank&log$=protalign&blast_rank=3&RID=30DY9FYK01N)  Length: 1117 aa | 1821 bits(4718) | 0.0 | 915/1129 (81%) | 1010/1129 (89%) | 16/1129 (1%) | Ascomycota |
| ***Metarhizium robertsii*** | [EXU98804.1](https://www.ncbi.nlm.nih.gov/protein/EXU98804.1?report=genbank&log$=protalign&blast_rank=4&RID=30DY9FYK01N)  Length: 1112 aa | 1821 bits(4716) | 0.0 | 911/1115 (82%) | 998/1115 (89%) | 17/1115 (1%) | Ascomycota |
| ***Fusarium avenaceum*** | [KAG5656294.1](https://www.ncbi.nlm.nih.gov/protein/KAG5656294.1?report=genbank&log$=protalign&blast_rank=5&RID=30GBHEXC016)  Length: 1116 aa | 1787 bits(4626) | 0.0 | 898/1104 (81%) | 995/1109 (90%) | 16/1104 (1%) | Ascomycota |
| ***Fusarium graminearum***  **PH-1** | [XP_011319222.1](https://www.ncbi.nlm.nih.gov/protein/XP_011319222.1?report=genbank&log$=protalign&blast_rank=7&RID=30GBHEXC016)  Length: 1114 aa | 1749 bits(4529) | 0.0 | 905/1129 (80%) | 1004/1129 (88%) | 19/1129 (1%) | Ascomycota |
| ***Histoplasma capsulatum*** | [KAG5304529.1](https://www.ncbi.nlm.nih.gov/protein/KAG5304529.1?report=genbank&log$=protalign&blast_rank=8&RID=30H2YBVM016)  Length: 1132 aa | 1682 bits(4356) | 0.0 | 825/1141 (72%) | 943/1141 (82%) | 26/1141 (2%) | Ascomycota |
| ***Aspergillus fumigatus* Af293** | [XP_751485.1](https://www.ncbi.nlm.nih.gov/protein/XP_751485.1?report=genbank&log$=protalign&blast_rank=12&RID=30H2YBVM016)  Length: 1111 aa | 1671 bits(4331) | 0.0 | 822/1085 (76%) | 921/1085 (84%) | 16/1085 (1%) | Ascomycota |
| ***Geotrichum candidum*** | [CDO54671.1](https://www.ncbi.nlm.nih.gov/protein/CDO54671.1?report=genbank&log$=protalign&blast_rank=1&RID=30JS6GS9016)  Length: 1107 aa | 1259 bits(3259) | 0.0 | 619/967 (64%) | 761/967 (78%) | 23/967 (2%) | Ascomycota |
| **Candida anglica** | [CAK7901703.1](https://www.ncbi.nlm.nih.gov/protein/CAK7901703.1?report=genbank&log$=protalign&blast_rank=12&RID=30JS6GS9016)  Length: 1055 aa | 1145 bits(2962) | 0.0 | 597/1023 (58%) | 740/1023 (78%) | 63/1023 (6%) | Ascomycota |
| ***Saccharomyces cerevisiae* YJM1402** | [AJU03976.1](https://www.ncbi.nlm.nih.gov/protein/AJU03976.1?report=genbank&log$=protalign&blast_rank=1&RID=30KGH8HG013)  Length: 1120 aa | 1148 bits(2970) | 0.0 | 575/1003 (57%) | 575/1003 (57%) | 57/1003 (5%) | Ascomycota |
| ***Ustilago* sp. UG-2017a** | [SOV09364.1](https://www.ncbi.nlm.nih.gov/protein/SOV09364.1?report=genbank&log$=protalign&blast_rank=1&RID=30MW8FFM01N)  Length: 1112 aa | 1142 bits(2955) | 0.0 | 569/985 (58%) | 735/985 (58%) | 45/985 (4%) | Basidiomycota |
| ***Rhizoctonia solani*** | [KAF8671695.1](https://www.ncbi.nlm.nih.gov/protein/KAF8671695.1?report=genbank&log$=protalign&blast_rank=2&RID=30MW8FFM01N)  Length: 1105 aa | 1128 bits(2918) | 0.0 | 587/1039 (56%) | 743/1039 (71%) | 51/1039 (4%) | Basidiomycota |
| ***Rhodotorula toruloides*** | [KAK4334512.1](https://www.ncbi.nlm.nih.gov/protein/KAK4334512.1?report=genbank&log$=protalign&blast_rank=16&RID=30NWNF0F013)  Length: 1156 aa | 1093 bits(2828) | 0.0 | 561/1042 (54%) | 718/1042 (68%) | 64/1042 (6%) | Basidiomycota |
| ***Armillaria mellea*** | [KAK0193940.1](https://www.ncbi.nlm.nih.gov/protein/KAK0193940.1?report=genbank&log$=protalign&blast_rank=25&RID=30NWNF0F013)  Length: 1074 aa | 1045 bits(2701) | 0.0 | 539/972 (55%) | 704/972 (72%) | 40/972 (4%) | Basidiomycota |
| ***Homarus americanus*** | [XP_042214026.1](https://www.ncbi.nlm.nih.gov/protein/XP_042214026.1?report=genbank&log$=protalign&blast_rank=2&RID=30PWWFDP016)  Length: 1030 aa | 1022 bits(2643) | 0.0 | 531/1006 (53%) | 679/1006 (67%) | 58/1006 (5%) | Animals |
| ***Gallus gallus* SMARCA5** | [XP_004941010.1](https://www.ncbi.nlm.nih.gov/protein/XP_004941010.1?report=genbank&log$=protalign&blast_rank=1&RID=30S6M94Y016)  Length: 995 aa | 990 bits(2559) | 0.0 | 510/962 (53%) | 659/962 (68%) | 43/962 (4%) | Animals |
| ***Mus musculus* SMARCA1** | [NP_001345548.1](https://www.ncbi.nlm.nih.gov/protein/NP_001345548.1?report=genbank&log$=protalign&blast_rank=4&RID=30SN8VGU013)  Length: 1046 aa | 974 bits(2519 | 0.0 | 487/952 (51%) | 640/952 (67%) | 33/952 (3%) | Animals |
| ***Homo sapiens* SMARCA5** | [NP_003592.3](https://www.ncbi.nlm.nih.gov/protein/NP_003592.3?report=genbank&log$=protalign&blast_rank=24&RID=30SN8VGU013)  Length: 1052 aa | 927 bits(2397) | 0.0 | 488/956 (51%) | 635/956 (66%) | 31/956 (3%) | Animals |
| ***Homo sapiens* SMARCA1** | [NP_001365191.1](https://www.ncbi.nlm.nih.gov/protein/NP_001365191.1?report=genbank&log$=protalign&blast_rank=2&RID=3G9SYAEU016)  Length: 1042 aa | 947 bits(2449) | 0.0 | 489/958 (51%) | 645/958 (67%) | 35/958(3%) | Animals |
| ***Dictyostelium discoideum* AX4** | [XP_629432.1](https://www.ncbi.nlm.nih.gov/protein/XP_629432.1?report=genbank&log$=protalign&blast_rank=1&RID=30TUEU7X016)  Length: 1221 aa | 797 bits(2058) | 0.0 | 449/993 (45%) | 612/993 (61%) | 63/993 (6%) | Protista |

**Figure S1A.** Similarity to MoIsw2 (*Magnaporthe oryzae* (teleomorph *Pyricularia oryzae*) XP_003717932 1128 aa. NCBI BLASTP using default settings. SMARCA1 and SMARCA5 labels to the Animals sequences are added since these 2 are the known ISW2 human orthologues with the abbreviation SMARCA (SWI/SNF related, matrix associated, actin dependent regulator of chromatin, subfamily a) often discussed in cancer research. They both have the same domain structures (Domains, and order of domains) but can form different complexes with other proteins.

**Figure S1B.** Alignment of protein sequences MoIsw2 in *Magnaporthe oryzae* (teleomorph *Pyricularia oryzae*).


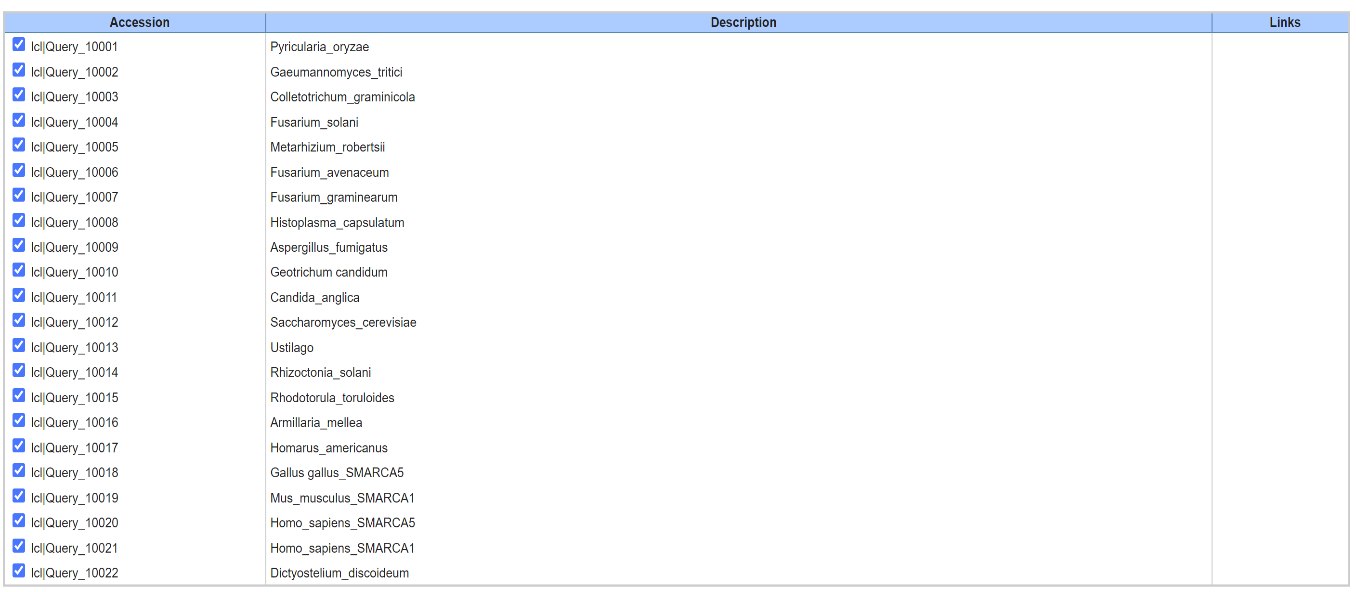

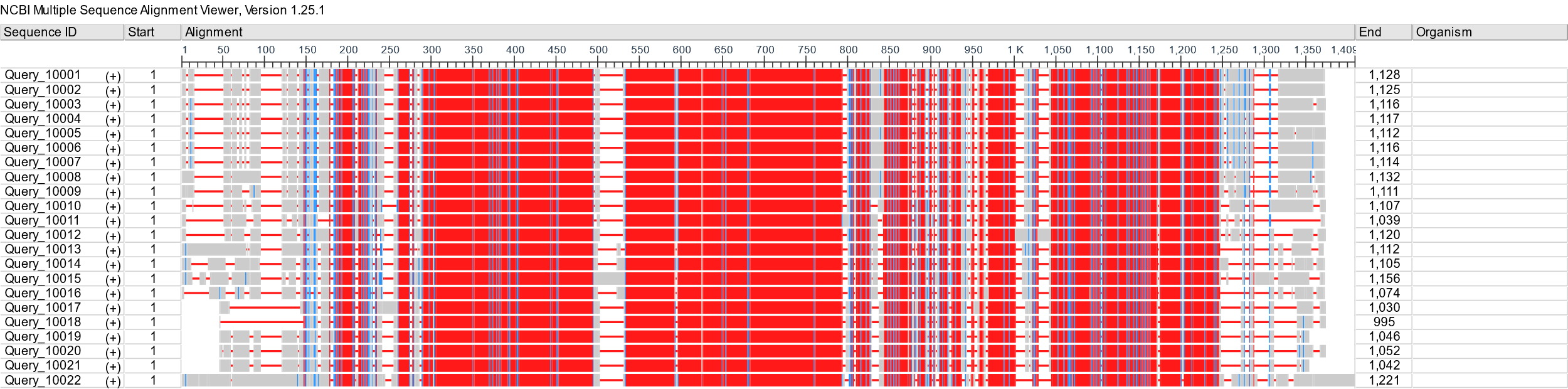


NCBI COBALT multiple alignment, Conservation coloring


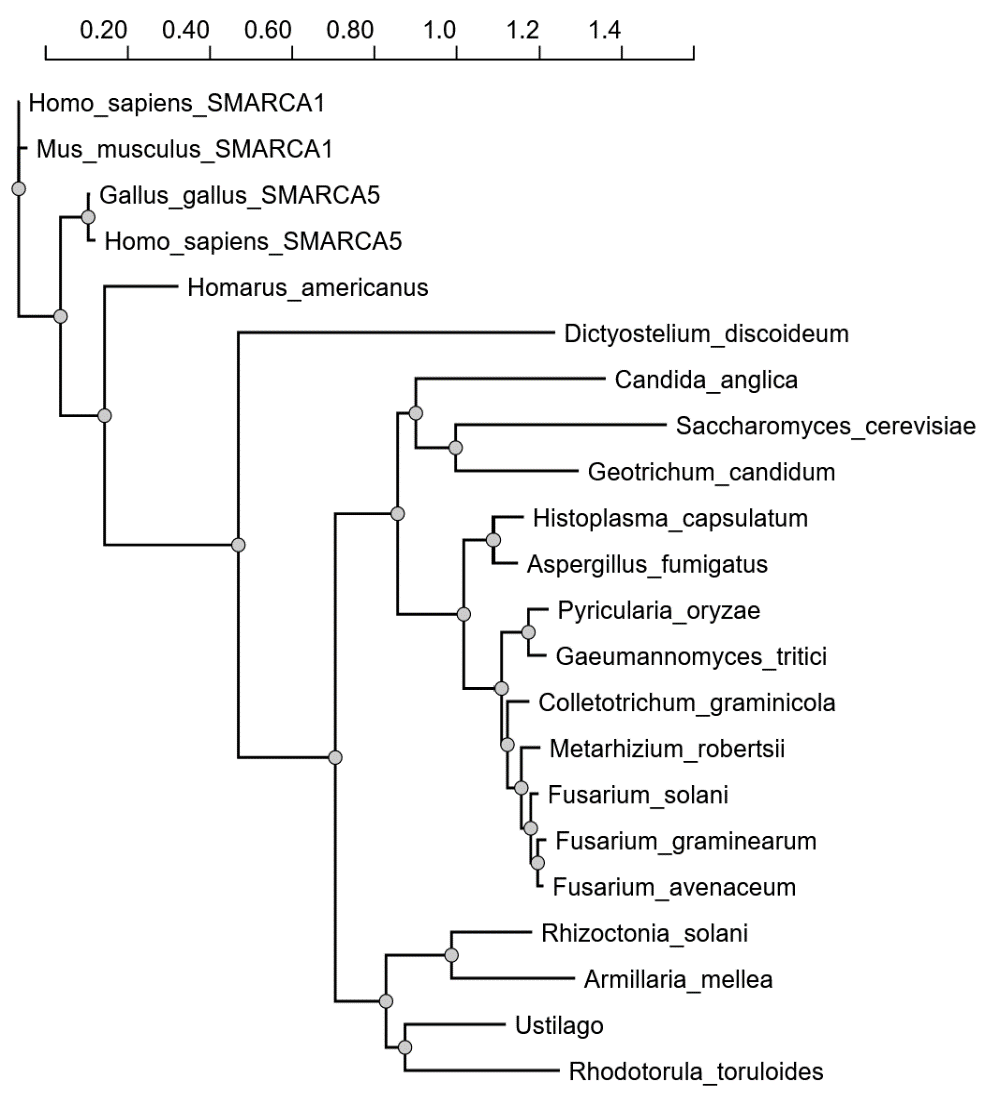


Phylogenetic tree

Ascomycota

Saccharomycotina

Basidiomycota

Protista

Animalia

Fungi

**Figure S2**. Phylogeny of MoIsw2 (*Magnaporthe oryzae* (teleomorph *Pyricularia oryzae*) compared to all sequences in Fig S2. See domain structures of the red-boxed species in Fig. S3. The tree was constructed using the 1-click option at NGPhylogeny.fr.

*Gallus gallus*


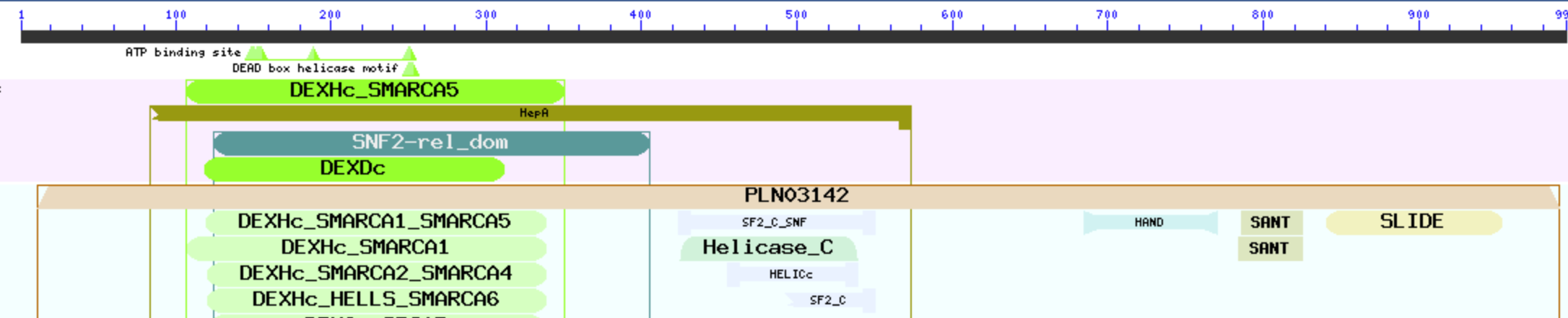


*Dictyostelium discoideum*


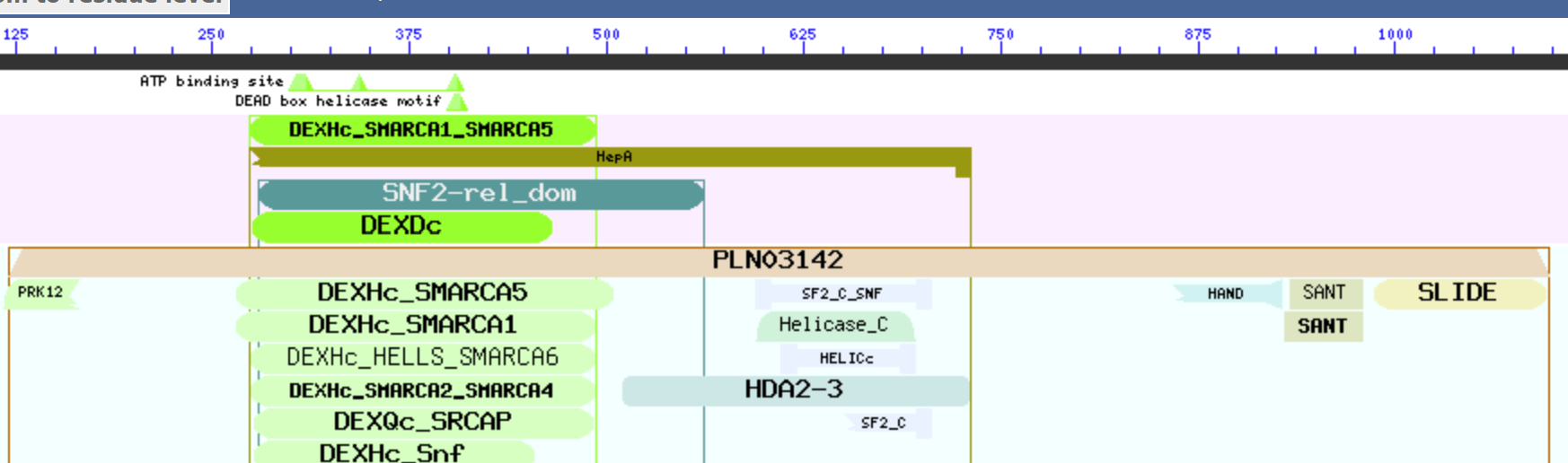


*Magnaporthe oryzae*


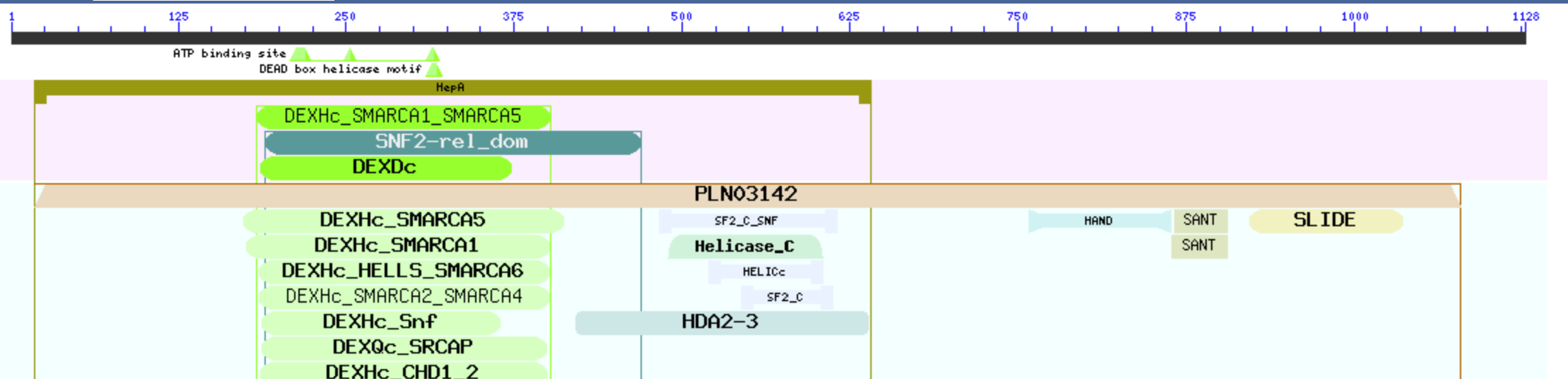


Saccharomyces cerevisiae


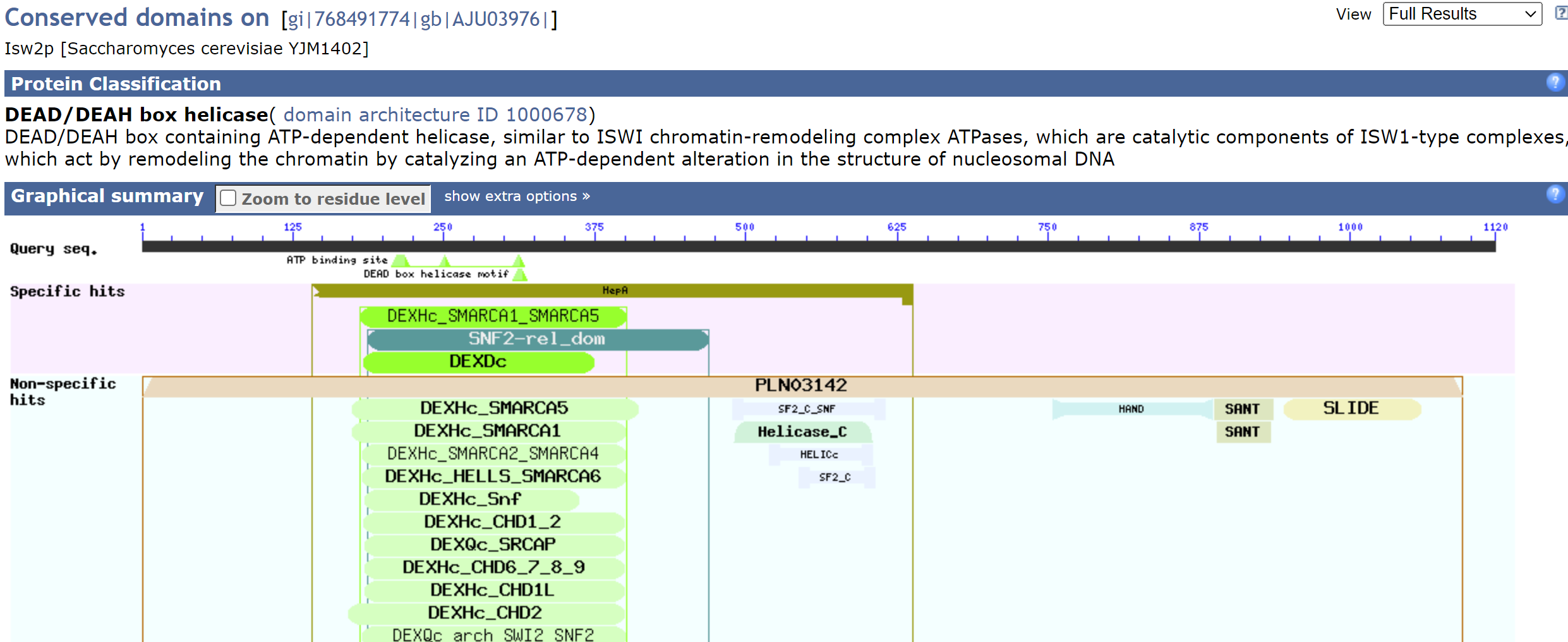


**Figure S3.** Domain structures of proteins are red-boxed in Fig. S2.


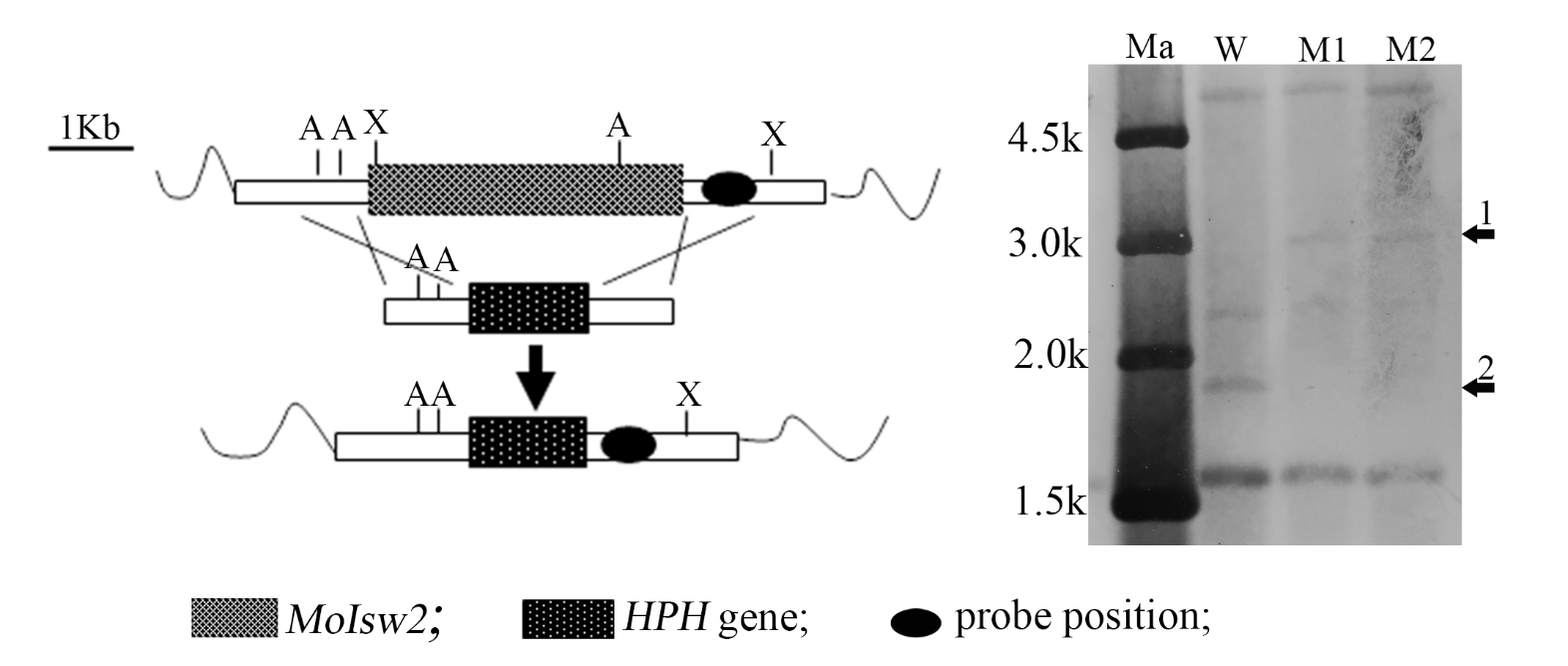


**Figure S4**. Southern blot confirmation for *MoISW2* mutants. The DNA sequence 1.0 kb downstream of the target gene was used as a probe for blotting. Arrow 1 indicates the expected bands that should be present in a successful mutant. Arrow 2 indicates bands that should be present in the background Ku80. W=Wildtype strain (Ku80), M1 and M2 two independent mutants, A= cleavage sites for *Age*I X= cleavage sites for *Xho*I.


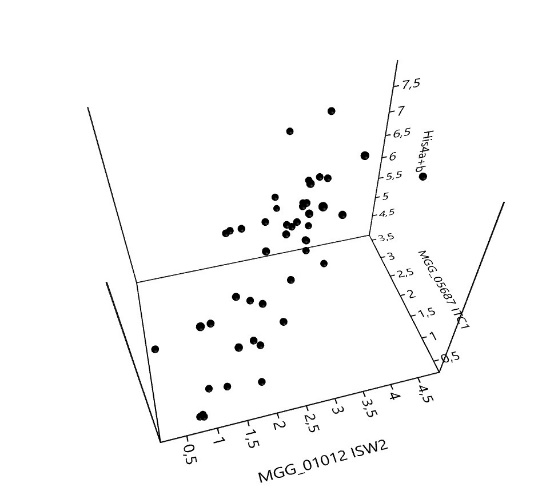


**Figure S5.** 3D plot showing the Log of expression of *MoISW2*, *MoITC1*, and *MoHIS4a+b* that, according to the literature, need to work together to influence nucleosome packing. They are all correlated and form a nice 3d “sausage” as they should if the three genes encode proteins that need to work together. Multiple regression with *MoISW2* as an independent variable, and *MoITC1*, and *MoHIS4a+b* dependent gives a linear relationship (P=6.7E-11).

**
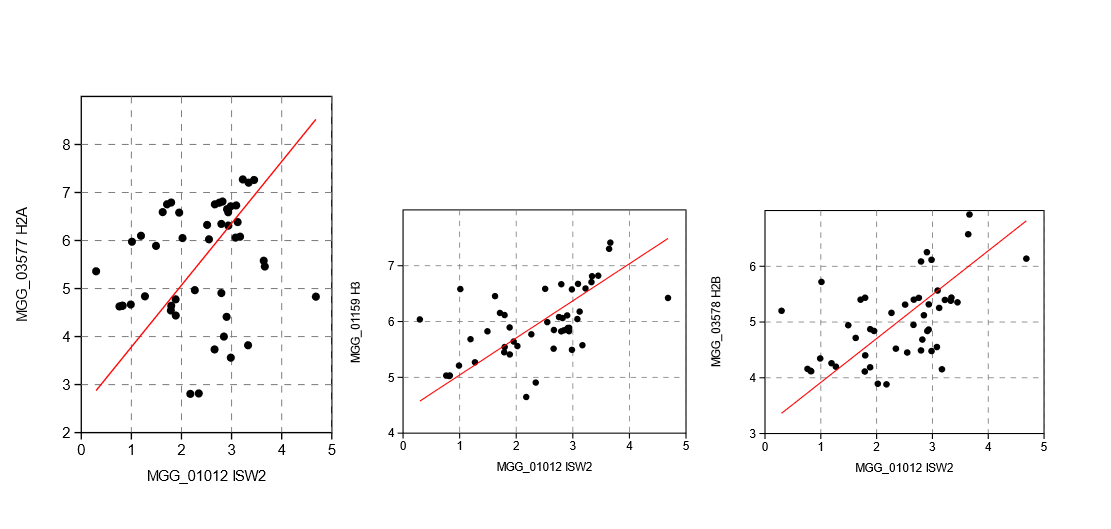
**

C

B

A

**Figure S6.** Log2 RMA correlations of a putative *MoHIS2a, MoHIS3* and *MoHIS2B* with the expression of *MoISW2* (x-axis) in published RNAseq data at different stages of plant infection. (**A**) *MoHIS2a*, P(uncorr=0.20 (**B**) *MoHIS3*, P(uncorr)=8.94E-5 (**C**) *MoHIS2B*, P(uncorr)=3.56E-5.

**Figure S7.** Comparison of the motif (Bottom) with the known human Myb protein DNA binding motif (Top) found by a TOMTOM query using the motif in Fig. 2F as the query. The MoIsw2 DNA binding site has a palindromic Myb/SANT-like DNA binding motif with a similarity to the human protein MYBL1.


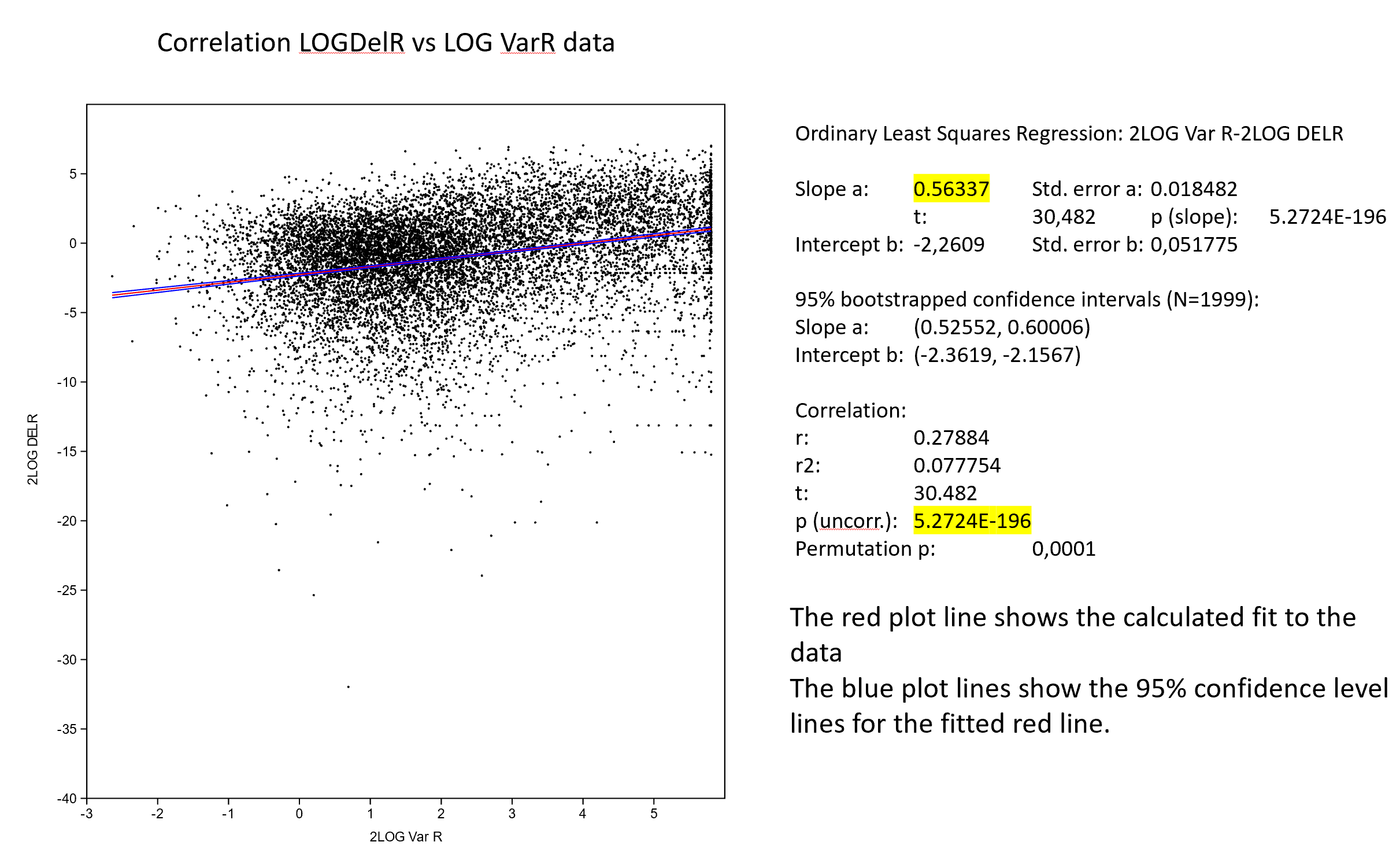


**Figure S8** Correlation between 2Log DelR vs 2Log VarR data. The VarR responses in planta and the DelR responses in vitro are correlated. Genes with very low VarR variability are good candidates as reference genes for the *in planta* experiments. Genes with low DelR variability are good candidates for the in vitro experiments. The commonly used reference gene that seemed to show very low variability was the actin gene. The technique should be able to be used to select good and stable reference gene candidates for RNAseq for different conditions. It should be able to find genes stable enough that small changes in the expression of target genes like TFs and other regulators and receptors can be detected since those gene classes can have large biological effects even with minor expression changes.


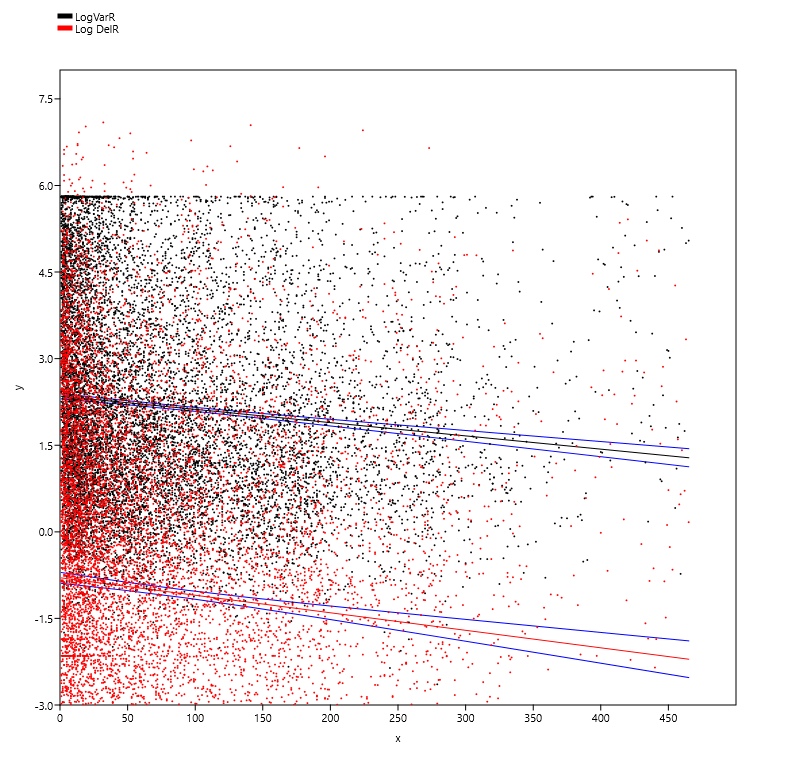

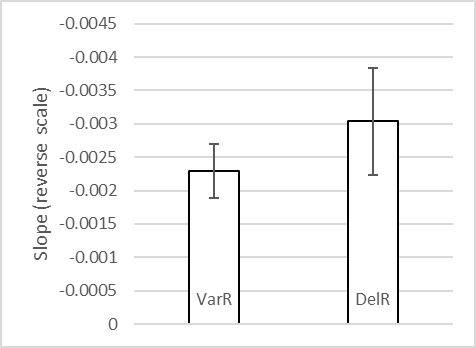


VarR, P (slope):

2.55E-25

DelR, P (slope):

2.03E-9

A

B

2Log Var

Gene distance from MoIsw2 binding in the Guy11 genome

**Figure S9**. Correlation of VarR and DelR with distance to closest MoIsw2 binding site in the Ku80 genome. (**A**) Both VarR and DelR are log-linear correlated and with non-significant differences in slopes. Blue lines are 95% confidence interval lines (**B**). Error bars in B show 95% confidence intervals for the bars’ mean slope values. These error bars overlap a lot indicating no significant difference

**Table S1. Primers used in this study**

| **Name** | **Sequences** | **Application** |
| --- | --- | --- |
| MGG_01012-UP-F | aagggaacaaaagctggtaccACGGCTTGACGGTTACTTGT | Gene deletion |
| MGG_01012-UP-R | tcagttaacgtcgacaagcttTTGTCGTGAGATTCCCTGGT | Gene deletion |
| MGG_01012-down-F | cgggaaccagttaacctgcagGTTTGGAACTCTTTGATGGG | Gene deletion |
| MGG_01012-down-R | cgctctagaactagtggatccGGGCTACTTTGACTTTATGT | Gene deletion |
| pCB1532-MGG_01012-Pro-F | cgctctagaactagtggatccAGGTGGATGATGTCGATTGCC | Gene complementation and protein localization |
| pCB1532-MGG_01012-EcoR1 | gcccttgctcaccatgaattcTTTCTTCTTGCCCTTGGCC | Gene complementation and protein localization |
| MGG_01012-TZ-F | gactttcgggacaagatgga | Southern blot probe |
| MGG_01012-TZ-R | acacccatcgcgaatagaac | Southern blot probe |
| pYES2-MGG_01012-Pro-F | taagcttggtaccgagctcggatccAGGTGGATGATGTCGATTGCC | Yeast scisw2 mutant complementation |
| pYES2-MGG_01012-EcoR1 | tgtgctggatatctgcagaattcTTTCTTCTTGCCCTTGGCC | Yeast scisw2 mutant complementation |

**Key resources table S2**

| REAGENT or RESOURCE | SOURCE | IDENTIFIER |
| --- | --- | --- |
| Chemicals, peptides, and recombinant proteins | | |
| MoISW2GFP | This paper |  |
| Critical commercial assays | | |
| ChIP seq service | IGENEBOOK  (see this paper supplemental file) | Supplemental Company methods.zip |
| RNAseq service | IGENEBOOK  (see this paper supplemental file) | Supplemental Company methods.zip |
| Deposited data | | |
| RNAseq compilation of published experiments on gene expressions during infection stages | Zhang et al.^2^ | <https://doi.org/10.6084/m9.figshare.7068857.v1> |
| ChIPsec data for MoISW2 binding M. oryzae Ku80 DNA  RNAseq data for strains ΔMoIsw2 and Ku80  and other **Supplemental data and analyses files 0-13 Supplemental company methods and Supplemental method 1** in a 50Mb zip file. | This paper | <https://doi.org/10.6084/m9.figshare.22218328> |
| *Magnaporthe oryzae* genome, genes sequences and gene contig order data needed to arrange genes in correct order on supercontigs. | BROAD institute | [ftp.broadinstitute.org/distribution/annotation/fungi/magnaporthe/genomes/magnaporthe_oryzae_70-15_8](ftp://ftp.broadinstitute.org/distribution/annotation/fungi/magnaporthe/genomes/magnaporthe_oryzae_70-15_8) User: Anonymous |
| Magnaporth oryzae expression Avir genes during rice infection. Strain 98-06 | Cao et al. ^3^ | <https://bsppjournals.onlinelibrary.wiley.com/action/downloadSupplement?doi=10.1111%2Fmpp.13224&file=mpp13224-sup-0010-TableS2.xlsx> |
| Sequence downloads, blasts, annotations including domain annotations | NCBI | https://www.ncbi.nlm.nih.gov/ |
| Experimental models: Organisms/strains | | |
| *Magnaporthe oryzae* (formerly *Magnaporthe grisea*) |  | Ku80^4^ deletion in strain Guy11 NCBI:txid242507 |
| MoISW2-GFP strain derived from Ku80 | Li et al. ^1^ | <https://doi.org/10.1101/2021.12.28.474317> |
| Software and algorithms | | |
| PAST statistics software package version 4.08 (released November 2021) | Hammer et al.^5^ | <https://www.nhm.uio.no/english/research/resources/past/> |
| Microsoft Excel (MS office 365) with Solver Add-In activated | Microsoft | <https://www.microsoft.com> |
| Fisher Exact Add-In for MS Excel | software@obertfamily.com | <http://www.obertfamily.com/software/fisherexact.html> |
|  |  |  |
|  |  |  |
| Other | | |
| FungiFun2 website for Functional Category analysis of putative proteins. | Priebe et al. ^6^ | <https://elbe.hki-jena.de/fungifun/fungifun.php> |
| antiSMASH website for finding core secondary metabolite genes in fungi. | Blin et al. ^7^ | [https://fungismash.secondarymetabolites.org/#!/start](https://fungismash.secondarymetabolites.org/%23!/start) |
| The MEME suite for use of MEME and FIMO | Bailey et al. ^8^ | <https://meme-suite.org/meme/> |

1. Li, Y. *et al.* *The* Myb *family genes in the rice pathogen* Magnaporthe oryzae*: Finding and deleting more family members involved in pathogenicity*. http://biorxiv.org/lookup/doi/10.1101/2021.12.28.474317 (2021) doi:10.1101/2021.12.28.474317.

2. Zhang, L. *et al.* Conserved Eukaryotic Kinase CK2 Chaperone Intrinsically Disordered Protein Interactions. *Appl Environ Microbiol* **86**, e02191-19, /aem/86/2/AEM.02191-19.atom (2019).

3. Cao, Y. *et al.* Characterization of two infection‐induced transcription factors of *Magnaporthe oryzae* reveals their roles in regulating early infection and effector expression. *Molecular Plant Pathology* **23**, 1200–1213 (2022).

4. Villalba, F. *et al.* Improved gene targeting in Magnaporthe grisea by inactivation of MgKU80 required for non-homologous end joining. *Fungal Genetics and Biology* **45**, 68–75 (2008).

5. Hammer, O., Harper, D. A. T. & Ryan, P. D. PAST: Paleontological Statistics Software Package for Education and Data Analysis. **4**, 9 (2001).

6. Priebe, S., Kreisel, C., Horn, F., Guthke, R. & Linde, J. FungiFun2: a comprehensive online resource for systematic analysis of gene lists from fungal species. *Bioinformatics* **31**, 445–446 (2015).

7. Blin, K. *et al.* antiSMASH 6.0: improving cluster detection and comparison capabilities. *Nucleic Acids Research* **49**, W29–W35 (2021).

8. Bailey, T. L., Johnson, J., Grant, C. E. & Noble, W. S. The MEME Suite. *Nucleic Acids Res* **43**, W39–W49 (2015).
